# Stress‐Related Brain Alterations in Chronic Pain

**DOI:** 10.1002/ejp.70034

**Published:** 2025-05-09

**Authors:** Yann Quidé, Negin Hesam‐Shariati, Nell Norman‐Nott, James H. McAuley, Sylvia M. Gustin

**Affiliations:** ^1^ NeuroRecovery Research Hub School of Psychology, UNSW Sydney Sydney New South Wales Australia; ^2^ Centre for Pain IMPACT Neuroscience Research Australia Randwick New South Wales Australia; ^3^ School of Health Sciences Faculty of Medicine and Health, UNSW Sydney Sydney New South Wales Australia

## Abstract

**Background:**

Stress symptoms are commonly experienced by people with chronic pain. Although stress and chronic pain are associated with similar effects on brain morphology, the present study aims to clarify the relationship between stress severity, chronic pain, and brain morphology.

**Methods:**

Fifty‐two people with chronic pain and 38 pain‐free healthy controls (HC) underwent T1‐weighted magnetic resonance imaging. Severity of stress symptoms was measured using the civilian version of the posttraumatic stress disorder checklist (PCL‐C). A series of multiple linear regressions determined the main effects of group, stress symptom severity (PCL‐C total score and symptom‐specific scores) and their interaction on grey matter volume of selected regions of interest.

**Results:**

The interaction term was significantly associated with variations in grey matter volume in the left and right putamen, the left middle cingulate cortex (MCC) and the right posterior insula. Results showed significantly smaller left and right putamen when reporting higher stress levels, and significantly larger left MCC and right posterior insula at lower stress levels in people with chronic pain compared to HCs. In addition, increasing stress severity was significantly associated with larger left and right putamen in HCs, and significantly associated with smaller left MCC and right posterior insula in people with chronic pain.

**Conclusions:**

Severity of stress moderated chronic pain‐related grey matter alterations. More severe stress, especially avoidance, was associated with smaller left MCC, a core region of the “pain matrix”. The MCC is strongly linked with the somatosensory network and critical for empathy, especially toward pain‐related stimuli.

**Significance:**

To our knowledge, this is the first study to show that stress severity differentially impacts grey matter integrity in people with chronic pain compared to pain‐free healthy controls. Briefly, our results indicate that higher levels of stress were associated with larger putamen and right posterior insula in pain‐free participants, potentially reflecting mechanisms of resilience to trauma in this group. Higher levels of stress, especially avoidance symptoms, were associated with smaller left middle cingulate cortex, a region with strong links to the somatosensory network and critical for empathy, especially toward pain‐related stimuli.

## 1 Introduction

Chronic pain is a major public health concern impacting around 28% of the global population (Zimmer et al. 2022), and often associated with severe comorbid mental health problems (Dominick et al. 2012), reduced quality of life (Hadi et al. 2019) and increased suicide risk (Tang and Crane 2006). Trauma exposure can trigger and contribute to the maintenance of pain through sensitisation of the stress system (Abdallah and Geha 2017). Chronic pain and trauma exposure are strongly related: around 20% of people with chronic pain experience posttraumatic stress symptoms (PTSS), when posttraumatic stress disorder (PTSD) is not formally diagnosed (Siqveland et al. 2017), and 20%–80% of people with PTSD experience chronic pain (Brennstuhl et al. 2015). However, the relationship between trauma and chronic pain is not completely understood.

The brain plays a key role in the development and maintenance of chronic pain (De Ridder et al. 2021). Reduced grey matter volume in the anterior cingulate cortex (ACC) and middle cingulate cortex (MCC), thalamus, insula, striatum, primary and secondary somatosensory cortices, part of the “pain matrix” (Melzack 1999; Tracey and Mantyh 2007), is commonly reported across chronic pain conditions (Neumann et al. 2023; Wang et al. 2022). Two subdivisions of the pain matrix manage different processes. The median matrix, made up of the ACC, prefrontal cortex, anterior insula, and thalamus, is dedicated to processing affective, motivational, and cognitive aspects of pain, whereas the lateral matrix, made up of the primary and secondary somatosensory cortices, posterior insula, and thalamus, processes the sensory‐discriminative aspects of pain (Moisset and Bouhassira 2007; Pricope et al. 2022). Alterations of these key brain regions are associated with comorbid depression, anxiety, and PTSD (Brandl et al. 2022; Quidé et al. 2023; Stevens and Jovanovic 2019; Zheng et al. 2022). Despite the high comorbidity between chronic pain and PTSD/PTSS, previous studies have focused on identifying the functional substrates of pain in PTSD (Choi et al. 2022; Seo et al. 2014), and the effects of PTSS on brain morphology in chronic pain, especially for regions that are part of the pain matrix and sensitive to stress/trauma, remain unknown.

Perhaps unsurprisingly, grey matter alterations in similar regions have been reported in independent PTSD and chronic pain studies, likely following dysregulation of common biological systems, including the neuroendocrine and immune systems (Abdallah and Geha 2017). Smaller hippocampus, ACC, insula, striatum, amygdala, middle frontal gyrus (MFG), and orbitofrontal/medial prefrontal cortex (mPFC) volumes are commonly reported brain alterations in PTSD (Logue et al. 2018; Meng et al. 2016; Siehl et al. 2023; Sun et al. 2022; Wang et al. 2021). Insular function is associated with hyper‐arousal and re‐experiencing symptoms (Stevens et al. 2018; Yehuda et al. 2015), while decreased ACC and increased insular function are associated with avoidance (Hopper et al. 2007; Paulus and Stein 2006). Despite morphological and functional differences (Uddin et al. 2017), few studies have separately investigated the anterior and posterior insulae in the context of trauma/PTSD and chronic pain (Harricharan et al. 2020). Overall, the relationship between stress/PTSS and morphological integrity of these regions in people with chronic pain remains largely unknown.

The present study aimed to clarify the relationship between chronic pain and stress/PTSS severity on brain morphology in key regions commonly reported in separate studies of PTSD and chronic pain: hippocampus, amygdala, striatum, thalamus, mPFC, MFG, ACC, MCC, and anterior and posterior insulae. We hypothesised that stress/PTSS severity will be associated with greater grey matter reductions in these key brain regions in people with chronic pain, compared to pain‐free healthy controls.

## 2 Methods and Materials

### 2.1 Participants

Participants from a convenience sample were 52 people reporting chronic pain conditions lasting for more than three months (together referred to as the *chronic pain* group), including temporomandibular disorder (TMD, *n* = 15), trigeminal neuropathic pain (TNP, *n* = 13), burning mouth (*n* = 1), trigeminal neuralgia (*n* = 6), TMD + TNP (*n* = 1), and spinal cord injury neuropathic pain (*n* = 16; complete paraplegia with continuous burning and/or shooting pain in areas of sensorimotor loss), as well as 38 pain‐free healthy controls (HC). Data collection for different studies using the same assessments and imaging protocols was performed by S.M.G. and pooled together by Y.Q. Individuals with chronic pain were recruited from specialised outpatient clinics, while pain‐free healthy controls were recruited through public advertisements. Inclusion criteria for all participants were age over 18 years old, and HCs reported no known diagnosis of psychiatric disorders. Neuropathic pain after spinal cord injury was diagnosed according to the International Association for the Study of Pain Spinal Cord Injury Pain Taxonomy (Bryce et al. 2012). All people with spinal cord injury suffered from a complete paraplegia with continuous burning and/or shooting pain in areas of sensorimotor loss. Painful TMD is characterised by ongoing musculoskeletal facial pain as assessed using the research diagnostic criteria for TMD (Dworkin and LeResche 1992). TNP and postherpetic neuralgia, which are both characterised by continuous dull neuropathic facial pain with sharp exacerbations, were diagnosed using the Liverpool Criteria (Nurmikko and Eldridge 2001). Exclusion criteria included having a heart pacemaker, metal implants, intrauterine contraceptive device, insulin pump, infusion devices, hearing disease, claustrophobia, pregnancy, a history of stroke, multiple sclerosis, or Parkinson's disease. All participants were volunteers who provided informed consent according to procedures approved by the Human Research Ethics committees of the University of New South Wales (HC15206), the University of Sydney (HREC06287) and Northern Sydney Local Health District (1102‐066M).

### 2.2 Assessments

The civilian version of the PTSD Checklist (PCL‐C) (Weathers et al. 1993) is a standardised self‐report 17‐item questionnaire used to measure the severity and burden of PTSS. Participants indicate how much they have been bothered by a symptom over the past month using a 5‐point scale (1 = not at all, 5 = extremely). In this study, no provision of a formal PTSD diagnosis was intended, criterion A was not assessed (e.g., using the Life Event Checklist) and participants were not explicitly asked about their experience of specific traumatic events. Thus, interpretation will be made in the context of stressful, rather than posttraumatic stress, symptoms. The overall burden of stress symptoms (PCL‐C total score, ranging from 17 to 85) was first established in focal analyses, followed by scoring for specific stress symptoms measured by the PCL‐C, including re‐experiencing (cluster B; questions 1–5), avoidance (cluster C; questions 6–12) and hyperarousal symptoms (cluster D; questions 13–17). Severity of depressive symptoms was measured using the sum of all 21 items from the Beck Depression Inventory (BDI‐I total scores ranging from 0 to 63) (Beck et al. 1961), and the severity of state anxiety was assessed using the 20‐item State subscale (scores ranging from 20 to 80) from the State–Trait Anxiety Inventory (STAI) (Spielberger et al. 1983).

A visual analogue scale (VAS) was used to evaluate participant's pain intensity in two ways. First, participants reported their experienced levels of pain on a 10‐cm horizontal ruler (‘no pain’ = 0 cm mark; ‘worst pain imaginable’ = 10 cm mark) three times a day (morning, noon, and evening) during the week (7 days) preceding their visit to the scanner (the ‘pain diary’). Second, pain during the scanning session was rated as soon as the participant left the scanner (the ‘scan pain’) (Table 1).

**TABLE 1:** Sociodemographic and clinical characteristics of the studied cohort.

	HC (*N* = 38)	Chronic pain (*N* = 52)	Statistics *Welch*/*t*/χ^2^	df	*p*
Age, in years, mean (SD) [range]	45.69 (17.33) [20.2–81.9]	52.31 (13.31) [23.8–76.1]	−1.967	66.78	0.053
Sex, *n* (F/M)	20/18	34/18	1.488	1	0.278
PCL‐C Total score (SD) [range]	22.61 (6.34) [17–44]	29.62 (9.04) [17–48]	**−4.324**	**87.88**	**< 0.001**
PCL‐C Re‐experiencing score (SD) [range]	6.24 (1.79) [5–13]	7.35 (3.00) [5–17]	**−2.188**	**85.07**	**0.031**
PCL‐C Avoidance score (SD) [range]	9.32 (3.64) [7–21]	12.27 (3.78) [7–21]	**−3.719**	**88**	**< 0.001**
PCL‐C Hyper‐arousal score (SD) [range]	7.05 (1.77) [5–11]	10.00 (4.12) [5–19]	**−4.613**	**73.58**	**< 0.001**
BDI total score (SD) [range]^a^	3.74 (4.03) [0–18]	9.75 (6.17) [0–29]	**−5.388**	**81.45**	**< 0.001**
STAI State, mean (SD) [range]^b^	20.00 (7.57) [12–44]	23.81 (8.34) [12–47]	**−2.174**	**83**	**0.033**
Pain condition (TMD/TNP/BM/TRIG/TMD + TNP/SCI)	—	15/13/1/6/1/16	—	—	—
Pain duration, in years, mean (SD) [range]	—	10.70 (10.55) [1–42]	—	—	—
VAS pain diary, mean (SD) [range]	—	3.99 (2.17) [0–9]	—	—	—
VAS scan pain, mean (SD) [range]^c^	—	3.42 (2.19) [0–9]	—	—	—
Scanning sites (NeuRA/SVH)	24/14	39/13	1.466	1	0.251

*Note:* Significant group differences are in bold.

Abbreviations: BDI, Beck Depression Inventory; df, degrees of freedom; F/M, females/males; HC, healthy controls; NeuRA, Neuroscience Research Australia; Pain, individuals with chronic pain; SD, standard deviation; STAI, State/Trait Anxiety Inventory; SVH, St Vincent's Hospital Sydney; TMD/TNP/BM/TRIG/TMD+TNP/SCI, temporomandibular disorder/trigeminal neuropathic pain/burning mouth/trigeminal neuralgia/temporomandibular disorder+trigeminal neuropathic pain/spinal cord injury; VAS, visual analogue scale.

^a^ Data missing for 3 HC and 3 chronic pain participants.

^b^ Data missing for 1 HC and 4 chronic pain participants.

^c^ Data missing for 3 chronic pain participants.

### 2.3 Magnetic Resonance Imaging

Imaging data were acquired for all participants on Philips 3T Achieva TX scanners (Philips Healthcare, Best, The Netherlands) housed at Neuroscience Research Australia (Randwick, New South Wales, Australia; HC, *n* = 24, Chronic Pain, *n* = 39) or at St Vincent's Hospital (Darlinghurst, New South Wales, Australia; HC, *n* = 14, Chronic Pain, *n* = 13). Both scanners were equipped with eight‐channel head‐coils and used the same acquisition parameters to collect 3D T1‐weighted Magnetization Prepared Rapid Gradient Echo (MPRAGE) structural scans (repetition time = 5.6 ms, echo time = 2.5 ms, field of view = 250 × 250 × 174 mm, matrix 288 × 288, 200 sagittal slices, flip angle = 8°, voxel size 0.9 × 0.9 × 0.9 mm).

A radiologist reviewed all scans before releasing them to the study investigators. An additional visual inspection for gross artefacts and movements (presence of excessive ringing that would not allow identification of two adjacent brain regions) was followed by automated quality control using the Computational Anatomy Toolbox (CAT12.8.1_2043; http://dbm.neuro.uni‐jena.de/cat/index.html) for Statistical Parametric Mapping (SPM12 v7771; Wellcome Trust Centre for Neuroimaging, London, UK; http://www.fil.ion.ucl.ac.uk/spm) in MATLAB r2022a (Mathworks Inc., Sherborn, MA, USA). Structural scans were pre‐processed using the CAT12 default routine for voxel‐based morphometry (VBM; see https://neuro‐jena.github.io/cat12‐help/#process_details). Following these steps, an additional quality control on sample homogeneity was performed to ensure there were no outlier scans (Mahalanobis distance between mean correlations and weighted overall image quality significantly higher than the other scans). Grey matter (GM) images were smoothed with an 8 mm full width at half maximum Gaussian kernel. Finally, total intracranial (TIV), total GM, total white matter (WM) and total cerebrospinal fluid (CSF) volumes were extracted for each participant. Average grey matter volumes for the selected 26 regions of interest (ROIs; left and right hippocampus, amygdala, caudate, putamen, pallidum, nucleus accumbens, thalamus, mPFC, MFG, ACC, MCC, anterior and posterior insula) were extracted from the Neuromorphometrics atlas in CAT12 for focal analyses.

### 2.4 Harmonisation

Before conducting statistical analyses, individual pre‐processed images and extracted ROI values were harmonised using the python‐based neuroHarmonize tools (https://github.com/rpomponio/neuroHarmonize) (Pomponio et al. 2020). This approach uses empirical Bayes methods derived from the ‘ComBat’ R package (Johnson et al. 2007) to adjust whole‐brain statistical maps and MRI‐derived indices of brain morphometry (GMV, WMV, CSF, TIV and ROIs) for variations associated with scanning location in multi‐site MRI studies. Age, sex, group, and PCL‐C total scores were modelled as covariates during harmonisation to ensure neuroHarmonize does not remove the variance associated with these variables.

### 2.5 Statistical Analyses

A series of multiple linear regressions was performed to determine the main effects of group (HC versus chronic pain), severity of stress symptoms (PCL‐C total score) and their interaction (the product of group × mean‐centered PCL‐C total score), first on grey matter volume of each a priori ROI (one model for each ROI), and second on whole‐brain VBM maps. Age, sex, and (harmonised) TIV were added as covariates in all neuroimaging analyses.

For ROI analyses, only models surviving Bonferroni correction to account for the number of ROIs tested were considered (*p* = 0.05/26 = 1.92 × 10^−3^). Power analysis using G*Power v3.1.9.6 (Faul et al. 2009; Faul et al. 2007) indicated that a minimum of 79 participants was necessary (*F*(6,72) = 3.90, *λ* = 27.65) to achieve 80% power for detecting a large (*f* ^2^ = 0.35) effect for 6 predictors (Group, PCL‐C score, Group‐by‐PCL‐C interaction, age, sex, harmonised TIV) at *α* = 1.92 × 10^−3^, accounting for the number of ROIs tested. For the whole‐brain analysis in SPM12/CAT12, statistical significance was set at an initial uncorrected voxel‐wise threshold of *p* < 0.001, to which family‐wise error correction was applied to the cluster statistics (family‐wise error‐corrected *p*‐threshold, *pFWEc* < 0.05). When significant effects were detected, raw signal at the cluster peak was extracted for further analyses in R (v4.3.1) (R Core Team 2023) and RStudio (2023.6.2.561) (Posit Team 2023).

In case of significant interactions, moderation analyses were performed using the ‘interactions’ R package (v1.1.5) (Long 2021). Two sets of moderation analyses were performed separately on each ROI or on the extracted raw signal at the cluster peak (for whole‐brain analyses) as the dependent variable. In the first moderation analysis, the effects of group (independent variable) were tested at three levels of stress symptoms severity (PCL‐C total score; moderator): at 1 standard deviation (SD) below the average PCL‐C total score (low PCL‐C total score), at average PCL‐C total score, and at 1 SD above the average PCL‐C total score (high PCL‐C total score) (Cohen et al. 2003). In the second moderation analysis, the effects of PCL‐C total score (independent variable) on indices of grey matter volume were tested for each group (moderator). The Davidson–MacKinnon correction (HC3) was used to account for potential issues related to heteroskedasticity (Hayes and Cai 2007) using the R package ‘sandwich’ (v3.2.2) (Zeileis 2004; Zeileis et al. 2020). Within each significant model, statistical significance was set at a threshold of *p* < 0.05.

### 2.6 Exploratory Analyses

To determine whether a specific symptom was driving the observed effects, exploratory follow‐up analyses were conducted on significant models using scores for the re‐experiencing, avoidance, and hyperarousal symptoms. Additional Bonferroni correction was applied to the original corrected threshold for significance to account for the number of symptoms studied for the ROIs (*p* = 1.92 × 10^−3^/3 = 6.41 × 10^−4^) and for the VBM maps (*pFWEc* = 0.05/3 = 0.017). Exploratory post hoc analyses were also performed within the groups of people with neuropathic and non‐neuropathic pain (TMD) separately to determine if some effects may be driven by specific types of pain (see Supporting Information Tables S2 and S3).

## 3 Results

### 3.1 Participant Characteristics

Demographic details are summarised in Table 1. Participants with chronic pain were not statistically different from the HC group in terms of age, sex, and scanning site distributions. However, they reported more severe stress symptoms, depression, and anxiety than the HC group. Pain intensity reported with the pain diary (average of three daily measures across seven days before the scanning session) was relatively low (mean = 3.99, standard deviation = 2.17), as was the pain intensity experienced during the scanning session (mean = 3.42, standard deviation = 2.19).

#### 3.1.1 ROI Analyses

**FIGURE 1:**
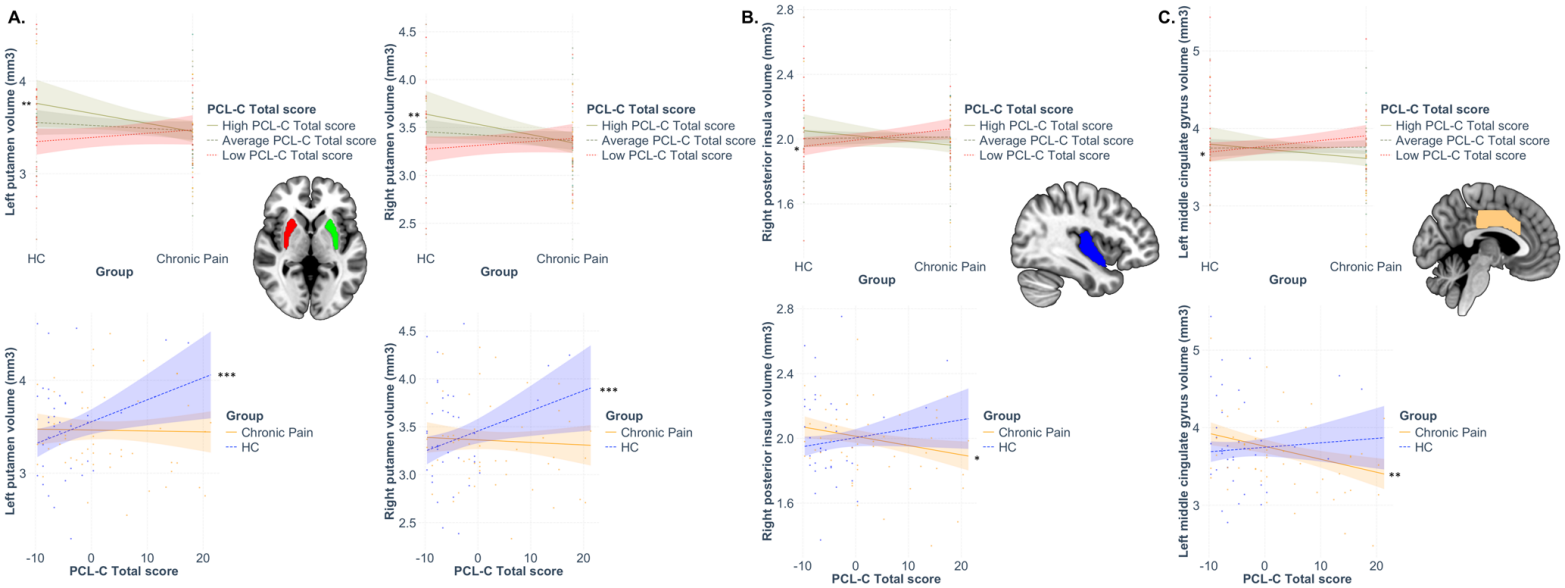
Moderation analyses following significant association between the group‐by‐PTSS total score interaction term and grey matter volume. The interaction term was significantly associated with variations in grey matter volume of the left and right putamen, the right posterior insula and the left middle cingulum cortex (MCC). (A) When using the PCL‐C total score as moderator of the group difference on left and right putamen volumes, analyses indicated that HCs had significantly larger left and right putamen compared to people with chronic pain, only at high levels of PTSS (brown plain lines). When using group as the moderator of the relationship between variations in PCL‐C scores and ROIs grey matter volumes, indicated that increasing levels of trauma were significantly associated with larger left and right putamen in the HC group only (blue dashed lines). (B) When using the PCL‐C total score as moderator of the group difference on right posterior insula volumes, analyses indicated that people with chronic pain had significantly larger right posterior insula compared to the HC group only at low levels of PTSS (red dashed lines). When using group as the moderator, increasing PCL‐C scores were associated with smaller right posterior insula in people with chronic pain only (yellow plain line). (C) When using the PCL‐C total score as moderator of the group difference on left MCC volume, analyses indicated that people with chronic pain had significantly larger left MCC than HCs (red dashed line). When using group as the moderator, increasing PCL_C scores were associated with smaller left MCC (yellow plain line). *p < 0.05; **p < 0.01; ***p < 0.001. Coloured band around each line represents 95% confidence intervals.

Table 2 summarises the results of all tested statistical models. All models, except those for the left and right pallidum and the left thalamus, were significant (*p* < 1.92 × 10^−3^). Of those, the group‐by‐trauma interaction was significantly associated with variations in grey matter volume in the left and right putamen, the left MCC, and the right posterior insula; this was also in the context of significant direct effects of stress symptoms severity on the left and right putamen (see Figure 1). The first moderation analysis using PCL‐C total score as moderator indicated that HCs had significantly larger left and right putamen compared to people with chronic pain, only at high levels of stress symptoms (but not at low or average levels). In addition, people with chronic pain had significantly larger left MCC and right posterior insula compared to the HC group only at low levels of stress symptoms (not at average or high levels). The second moderation analysis using group as the moderator of the relationship between variations in PCL‐C scores and ROIs grey matter volumes indicated that increasing levels of stress were significantly associated with larger left and right putamen in the HC group only, and with smaller left MCC and right posterior insula in people with chronic pain only.

**TABLE 2:** Results of the moderation analyses for all ROIs.

ROI	Model				Group						Stress (PCL‐C total score)						Group × Stress					
	*Adj R* ^2^	*F*	df	*p*	*b*	*se*	LLCI	ULCI	*t*‐value	*p*	*b*	*se*	LLCI	ULCI	*t*‐value	*p*	*b*	*se*	LLCI	ULCI	*t*‐value	*p*
LNAcc	0.561	16.473	6, 83	**< 1.92 × 10–3**	−0.0154	0.0097	−0.0346	0.0038	−1.5935	0.1148	0.0004	0.0011	−0.0018	0.0026	0.3502	0.7271	−0.0010	0.0012	−0.0034	0.0014	−0.8241	0.4122
RNAcc	0.571	16.532	6, 83	**< 1.92 × 10–3**	−0.0072	0.0078	−0.0228	0.0083	−0.9243	0.3580	0.0002	0.0009	−0.0017	0.0020	0.1646	0.8697	−0.0010	0.0010	−0.0030	0.0010	−1.0036	0.3185
LCaud	0.536	17.095	6, 83	**< 1.92 × 10–3**	−0.0589	0.0669	−0.1920	0.0742	−0.8798	0.3815	0.0062	0.0070	−0.0077	0.0201	0.8849	0.3788	−0.0040	0.0080	−0.0199	0.0118	−0.5063	0.6140
RCaud	0.488	12.755	6, 83	**< 1.92 × 10–3**	−0.0626	0.0738	−0.2093	0.0841	−0.8486	0.3986	0.0033	0.0075	−0.0115	0.0182	0.4459	0.6569	−0.0028	0.0086	−0.0198	0.0143	−0.3215	0.7486
LPut	0.505	18.111	6, 83	**< 1.92 × 10–3**	−0.0887	0.0788	−0.2454	0.0680	−1.1263	0.2633	**0.0237**	**0.0065**	**0.0108**	**0.0366**	**3.6515**	**0.0005**	**−0.0247**	**0.0088**	**−0.0422**	**−0.0071**	**−2.7958**	**0.0064**
															**HC**		**0.0237**	**0.0065**	**0.0108**	**0.0366**	**3.6515**	**0.0005**
															Chronic Pain		−0.0010	0.0059	−0.0127	0.0108	−0.1677	0.8672
															Low Trauma		0.1259	0.1056	−0.0842	0.3360	1.1919	0.2367
															Average Trauma		−0.0887	0.0788	−0.2454	0.0680	−1.1263	0.2633
															**High Trauma**		**−0.3034**	**0.1142**	**−0.5304**	**−0.0763**	**−2.6570**	**0.0095**
RPut	0.529	26.424	6, 83	**< 1.92 × 10–3**	−0.0927	0.0736	−0.2392	0.0537	−1.2595	0.2114	**0.0210**	**0.0054**	**0.0102**	**0.0319**	**3.8600**	**0.0002**	**−0.0237**	**0.0080**	**−0.0395**	**−0.0078**	**−2.9713**	**0.0039**
															**HC**		**0.0210**	**0.0054**	**0.0102**	**0.0319**	**3.8600**	**0.0002**
															Chronic Pain		−0.0027	0.0055	−0.0136	0.0083	−0.4831	0.6303
															Low Trauma		0.1132	0.1108	−0.1072	0.3336	1.0215	0.3100
															Average Trauma		−0.0927	0.0736	−0.2392	0.0537	−1.2595	0.2114
															**High Trauma**		**−0.2987**	**0.0904**	**−0.4785**	**−0.1189**	**−3.3042**	**0.0014**
LPallid	0.041	1.403	6.83	0.2231	−0.0025	0.0232	−0.0486	0.0436	−0.1062	0.9157	0.0005	0.0016	−0.0027	0.0037	0.3016	0.7637	0.0005	0.0022	−0.0039	0.0049	0.2222	0.8247
RPallid	0.066	1.634	6, 83	0.1479	0.0010	0.0224	−0.0436	0.0455	0.0431	0.9657	−0.0003	0.0015	−0.0032	0.0026	−0.2066	0.8368	0.0012	0.0022	−0.0031	0.0055	0.5437	0.5881
LAmyg	0.688	34.634	6, 83	**< 1.92 × 10–3**	−0.0154	0.0153	−0.0457	0.0150	−1.0083	0.3162	0.0013	0.0016	−0.0019	0.0046	0.8261	0.4111	−0.0035	0.0019	−0.0072	0.0003	−1.8479	0.0682
RAmyg	0.733	40.606	6, 83	**< 1.92 × 10–3**	−0.0033	0.0148	−0.0327	0.0261	−0.2218	0.8250	0.0002	0.0017	−0.0031	0.0036	0.1390	0.8898	−0.0018	0.0019	−0.0057	0.0021	−0.9255	0.3574
LHippo	0.571	18.195	6, 83	**< 1.92 × 10–3**	−0.0783	0.0652	−0.2079	0.0514	−1.2007	0.2333	0.0019	0.0068	−0.0117	0.0154	0.2769	0.7825	0.0019	0.0081	−0.0143	0.0181	0.2347	0.8150
RHippo	0.596	24.072	6, 83	**< 1.92 × 10–3**	−0.0414	0.0663	−0.1733	0.0905	−0.6238	0.5345	0.0009	0.0077	−0.0144	0.0162	0.1142	0.9093	0.0033	0.0092	−0.0149	0.0216	0.3640	0.7168
LThal	0.199	3.975	6, 83	0.0022	0.0873	0.1238	−0.1588	0.3335	0.7057	0.4823	−0.0071	0.0152	−0.0373	0.0230	−0.4705	0.6392	0.0028	0.0175	−0.0320	0.0376	0.1589	0.8741
RThal	0.208	5.280	6, 83	**< 1.92 × 10–3**	0.2175	0.1422	−0.0652	0.5003	1.5302	0.1298	−0.0089	0.0156	−0.0399	0.0221	−0.5723	0.5687	0.0073	0.0175	−0.0274	0.0421	0.4209	0.6749
LMFC	0.616	28.479	6, 83	**< 1.92 × 10–3**	−0.0224	0.0494	−0.1207	0.0758	−0.4543	0.6508	−0.0012	0.0058	−0.0127	0.0103	−0.2054	0.8378	0.0033	0.0063	−0.0094	0.0159	0.5130	0.6093
RMFC	0.644	34.295	6, 83	**< 1.92 × 10–3**	−0.0563	0.0492	−0.1541	0.0415	−1.1452	0.2554	−0.0012	0.0059	−0.0130	0.0106	−0.2053	0.8378	0.0033	0.0066	−0.0097	0.0163	0.5027	0.6165
LAntCgG	0.691	32.385	6, 83	**< 1.92 × 10–3**	−0.2111	0.1062	−0.4223	0.0001	−1.9881	0.0501	0.0088	0.0120	−0.0151	0.0326	0.7300	0.4674	−0.0138	0.0132	−0.0402	0.0125	−1.0457	0.2987
RAntCgG	0.564	17.108	6, 83	**< 1.92 × 10–3**	−0.0190	0.1243	−0.2661	0.2282	−0.1527	0.8790	0.0112	0.0157	−0.0200	0.0424	0.7151	0.4766	−0.0182	0.0164	−0.0509	0.0145	−1.1092	0.2705
LMCgG	0.700	34.295	6, 83	**< 1.92 × 10–3**	0.0128	0.0797	−0.1457	0.1712	0.1601	0.8732	0.0058	0.0092	−0.0125	0.0240	0.6303	0.5302	**−0.0225**	**0.0104**	**−0.0433**	**−0.0017**	**−2.1556**	**0.0340**
															HC		0.0058	0.0092	−0.0125	0.0240	0.6303	0.5302
															**Chronic Pain**		**−0.0167**	**0.0053**	**−0.0274**	**−0.0061**	**−3.1302**	**0.0024**
															**Low Trauma**		**0.2086**	**0.0998**	**0.0101**	**0.4071**	**2.0905**	**0.0396**
															Average Trauma		0.0128	0.0797	−0.1457	0.1712	0.1601	0.8732
															High Trauma		−0.1831	0.1387	−0.4591	0.0928	−1.3202	0.1904
RMCgG	0.675	38.136	6, 83	**< 1.92 × 10–3**	0.0601	0.1159	−0.1704	0.2905	0.5184	0.6055	0.0049	0.0153	−0.0254	0.0353	0.3223	0.7480	−0.0184	0.0156	−0.0494	0.0126	−1.1791	0.2417
LMFC	0.736	28.572	6, 83	**< 1.92 × 10–3**	−0.3965	0.3077	−1.0085	0.2156	−1.2883	0.2012	0.0309	0.0348	−0.0384	0.1002	0.8872	0.3776	−0.0536	0.0411	−0.1354	0.0283	−1.3019	0.1965
RMFG	0.778	43.404	6, 83	**< 1.92 × 10–3**	**−0.5237**	**0.2395**	**−1.0000**	**−0.0474**	**−2.1869**	**0.0316**	0.0344	0.0205	−0.0063	0.0751	1.6791	0.0969	−0.0382	0.0256	−0.0892	0.0128	−1.4913	0.1397
LAIns	0.656	16.398	6, 83	**< 1.92 × 10–3**	−0.0786	0.0699	−0.2177	0.0604	−1.1247	0.2639	**0.0159**	**0.0074**	**0.0012**	**0.0306**	**2.1554**	**0.0340**	−0.0137	0.0094	−0.0323	0.0049	−1.4655	0.1466
RAIns	0.623	15.335	6, 83	**< 1.92 × 10–3**	−0.0170	0.0667	−0.1496	0.1156	−0.2552	0.7992	0.0109	0.0061	−0.0013	0.0231	1.7829	0.0783	−0.0095	0.0081	−0.0257	0.0066	−1.1704	0.2452
LPIns	0.705	28.602	6, 83	**< 1.92 × 10–3**	0.0316	0.0371	−0.0422	0.1053	0.8515	0.3970	0.0040	0.0042	−0.0043	0.0122	0.9556	0.3421	−0.0074	0.0049	−0.0170	0.0023	−1.5118	0.1344
RPIns	0.711	40.855	6, 83	**< 1.92 × 10–3**	0.0092	0.0403	−0.0710	0.0893	0.2280	0.8202	0.0055	0.0049	−0.0042	0.0153	1.1265	0.2632	**−0.0113**	**0.0054**	**−0.0219**	**−0.0006**	**−2.0962**	**0.0391**
															HC		0.0055	0.0049	−0.0042	0.0153	1.1265	0.2632
															**Chronic Pain**		**−0.0057**	**0.0023**	**−0.0104**	**−0.0011**	**−2.4550**	**0.0162**
															**Low Trauma**		**0.1071**	**0.0436**	**0.0205**	**0.1937**	**2.4586**	**0.0160**
															Average Trauma		0.0092	0.0403	−0.0710	0.0893	0.2280	0.8202
															High Trauma		−0.0887	0.0756	−0.2390	0.0616	−1.1737	0.2439

*Note:* Statistically significant associations (*p* < 0.05 within each model) are in bold and highlighted in grey.

Abbreviations: Adj *R* ^2^, adjusted coefficient of determination; AIns, anterior insula; Amyg, amygdala; AntCgG, anterior cingulate gyrus; Caud, caudate nucleus; Hippo, hippocampus; L/R, left/right; LLCI, bootstrapped 95% lower levels confidence interval; MCgG, middle cingulate gyrus; MFC, medial frontal cortex; MFG, middle frontal gyrus; NAcc, nucleus accumbens; Pallid, pallidum; PCLC, posttraumatic stress disorder checklist—civilian; Pins, posterior insula; Put, putamen; ROI, region of interest; se, standard error; Thal, thalamus proper; ULCI, bootstrapped 95% upper levels confidence interval.

In addition, there was a significant effect of group showing smaller right MFG volume in the chronic pain group relative to the HC group (independent of trauma severity), and a significant increase in volume of the left anterior insula in association with increasing stress symptom severity (independent of group).

#### 3.1.2 Whole‐Brain Analyses

There was no significant association between group, PCL‐C total score, or their interaction on the whole‐brain VBM maps.

#### 3.1.3 Exploratory Analyses

Exploratory analyses were conducted on the ROIs showing a significant association with the group‐by‐trauma interaction (using the PCL‐C total score) above; that is, the left and right putamen, the left MCC, and the right posterior insula. Details of these exploratory ROI analyses are presented in Table 3.

**TABLE 3:** Results of the moderation analyses with separate PTSS symptoms.

ROI	Model				Group						Stress (PCL‐C score)						Group × Stress					
	*Adj R* ^2^	*F*	df	*p*	*b*	*se*	LLCI	ULCI	*t*‐value	*p*	*b*	*se*	LLCI	ULCI	*t*‐value	*p*	*b*	*se*	LLCI	ULCI	*t*‐value	*p*
Re‐experiencing																						
LPut	0.514	18.870	6, 83	**< 1.92 × 10–3**	−0.0541	0.0752	−0.2037	0.0955	−0.7196	0.4738	**0.0918**	**0.0242**	**0.0437**	**0.1399**	**3.7925**	**0.0003**	**−0.0981**	**0.0337**	**−0.1651**	**−0.0311**	**−2.9111**	**0.0046**
															**HC**		**0.0918**	**0.0242**	**0.0437**	**0.1399**	**3.7925**	**0.0003**
															Chronic Pain		−0.0063	0.0218	−0.0497	0.0371	−0.2882	0.7739
															Low Trauma		0.2013	0.1138	−0.0251	0.4277	1.7686	0.0806
															Average Trauma		−0.0541	0.0752	−0.2037	0.0955	−0.7196	0.4738
															**High Trauma**		**−0.3096**	**0.1173**	**−0.5428**	**−0.0763**	**−2.6398**	**0.0099**
RPut	0.547	27.448	6, 83	**< 1.92 × 10–3**	−0.0711	0.0665	−0.2035	0.0612	−1.0692	0.2881	**0.0909**	**0.0203**	**0.0504**	**0.1313**	**4.4677**	**< 0.0001**	**−0.1023**	**0.0297**	**−0.1613**	**−0.0432**	**−3.4449**	**0.0009**
															**HC**		**0.0909**	**0.0203**	**0.0504**	**0.1313**	**4.4677**	**< 0.0001**
															Chronic Pain		−0.0114	0.0196	−0.0503	0.0275	−0.5837	0.5610
															Low Trauma		0.1952	0.1111	−0.0257	0.4162	1.7573	0.0825
															Average Trauma		−0.0711	0.0665	−0.2035	0.0612	−1.0692	0.2881
															**High Trauma**		**−0.3375**	**0.0921**	**−0.5206**	**−0.1544**	**−3.6665**	**0.0004**
LMCgG	0.661	29.822	6, 83	**< 1.92 × 10–3**	−0.0201	0.0771	−0.1734	0.1333	−0.2605	0.7951	0.0217	0.0338	−0.0454	0.0889	0.6436	0.5216	−0.0383	0.0372	−0.1123	0.0357	−1.0300	0.3060
RPIns	0.705	37.607	6, 83	**< 1.92 × 10–3**	0.0001	0.0344	−0.0683	0.0685	0.0025	0.9980	0.0286	0.0160	−0.0032	0.0604	1.7911	0.0769	**−0.0393**	**0.0185**	**−0.0760**	**−0.0025**	**−2.1267**	**0.0364**
															HC		0.0286	0.0160	−0.0032	0.0604	1.7911	0.0769
															Chronic Pain		−0.0107	0.0093	−0.0292	0.0078	−1.1474	0.2545
															**Low Trauma**		**0.1024**	**0.0477**	**0.0075**	**0.1972**	**2.1469**	**0.0347**
															Average Trauma		0.0001	0.0344	−0.0683	0.0685	0.0025	0.9980
															High Trauma		−0.1022	0.0687	−0.2388	0.0344	−1.4883	0.1405
Avoidance																						
LPut	0.508	19.251	6, 83	**< 1.92 × 10–3**	−0.081	0.0765	−0.2332	0.0713	−1.0579	0.2932	**0.0413**	**0.0116**	**0.0183**	**0.0644**	**3.5726**	**0.0006**	−0.0323	0.0192	−0.0705	0.0059	−1.6799	0.0967
RPut	0.528	25.195	6, 83	**< 1.92 × 10–3**	−0.0841	0.0749	−0.2331	0.0648	−1.1232	0.2646	**0.0359**	**0.0098**	**0.0163**	**0.0555**	**3.6455**	**0.0005**	−0.0318	0.0176	−0.0668	0.0031	−1.8132	0.0734
LMCgG	0.723	40.045	6, 83	**< 1.92 × 10–3**	0.0357	0.0747	−0.1129	0.1843	0.478	0.6339	0.0097	0.0145	−0.0191	0.0384	0.6693	0.5051	**−0.0578**	**0.0198**	**−0.0972**	**−0.0185**	**−2.9217**	**0.0045**
															HC		0.0097	0.0145	−0.0191	0.0384	0.6693	0.5051
															**Chronic Pain**		**−0.0481**	**0.014**	**−0.0759**	**−0.0204**	**−3.4465**	**0.0009**
															**Low Trauma**		**0.2658**	**0.1035**	**0.06**	**0.4716**	**2.5685**	**0.0120**
															Average Trauma		0.0357	0.0747	−0.1129	0.1843	0.478	0.6339
															High Trauma		−0.1944	0.1134	−0.4199	0.0312	−1.7139	0.0903
RPIns	0.707	38.033	6, 83	**< 1.92 × 10–3**	0.0217	0.0398	−0.0574	0.1008	0.5453	0.587	0.0064	0.0091	−0.0117	0.0246	0.704	0.4834	−0.0198	0.0104	−0.0405	0.0009	−1.9046	0.0603
Hyperarousal																						
LPut	0.474	13.152	6, 83	**< 1.92 × 10–3**	−0.0429	0.1115	−0.2646	0.1788	−0.3849	0.7013	0.0356	0.0347	−0.0334	0.1047	1.0260	0.3079	−0.0448	0.0357	−0.1158	0.0263	−1.2526	0.2139
RPut	0.502	17.140	6, 83	**< 1.92 × 10–3**	−0.0435	0.0996	−0.2416	0.1546	−0.4367	0.6635	0.0248	0.0308	−0.0365	0.0860	0.8034	0.4240	−0.0350	0.0321	−0.0989	0.0288	−1.0909	0.2785
LMCgG	0.685	34.885	6, 83	**< 1.92 × 10–3**	0.0144	0.0828	−0.1502	0.1790	0.1737	0.8626	0.0057	0.0303	−0.0547	0.0660	0.1866	0.8525	−0.0362	0.0320	−0.0998	0.0274	−1.1332	0.2604
RPIns	0.700	39.034	6, 83	**< 1.92 × 10–3**	0.0061	0.0411	−0.0756	0.0878	0.1490	0.8819	0.0132	0.0147	−0.0160	0.0423	0.8989	0.3713	−0.0239	0.0152	−0.0541	0.0064	−1.5707	0.1201

*Note:* Statistically significant associations (*p* < 0.05 within each model) are in bold and highlighted in grey.

Abbreviations: Adj *R* ^2^, adjusted coefficient of determination; L/R, left/right; LLCI, bootstrapped 95% lower levels confidence interval; MCgG, middle cingulate gyrus; PCLC, posttraumatic stress disorder checklist—civilian; Pins, posterior insula; Put, putamen; ROI, region of interest; se, standard error; ULCI, bootstrapped 95% upper levels confidence interval.

##### 3.1.3.1 Re‐Experiencing

**FIGURE 2:**
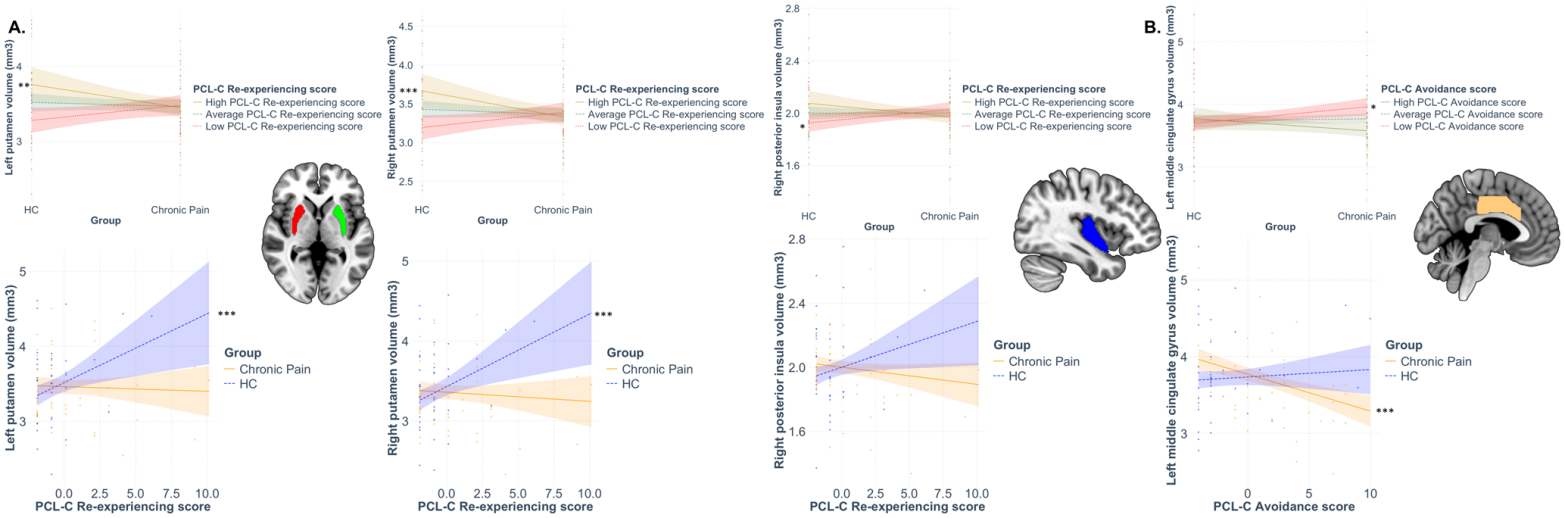
Moderation analyses following significant association between the group‐by‐PTSS interaction term and grey matter volume. (A) Re‐experiencing symptoms significantly moderated the group differences on left and right putamen and right posterior insula. Larger putamen and right posterior insula were evident in HCs compared to people with chronic pain at higher levels of re‐experiencing only (brown plain lines). In addition, increasing re‐experiencing symptoms were associated with larger left and right putamen only in HCs (blue dashed lines). (B) Avoidance symptoms significantly moderated the group differences on left MCC. Larger left MCC was evident in people with chronic pain compared to HCs at lower levels of avoidance only (red dashed lines). In addition, increasing avoidance symptoms were associated with smaller left MCC only in people with chronic pain (yellow plain lines). *p < 0.05; **p < 0.01; ***p < 0.001. Coloured band around each line represents 95% confidence intervals.

The group‐by‐trauma interaction was significantly associated with variations in the left and right putamen, and the right posterior insula, but not the left MCC (see Figure 2). The first moderation analysis using PCL‐C re‐experiencing score as a moderator showed significantly larger left and right putamen at high levels of trauma re‐experiencing (but not at low or average levels), and smaller right posterior insula at low levels of trauma re‐experiencing (not at average or high levels) in HCs relative to people with chronic pain. The second moderation analysis using group as the moderator of the relationship between variations in PCL‐C re‐experiencing scores and variations in grey matter volume of the ROIs indicated that larger left and right putamen were significantly associated with increasing levels of re‐experiencing in the HC group only.

##### 3.1.3.2 Avoidance

**FIGURE 3:**
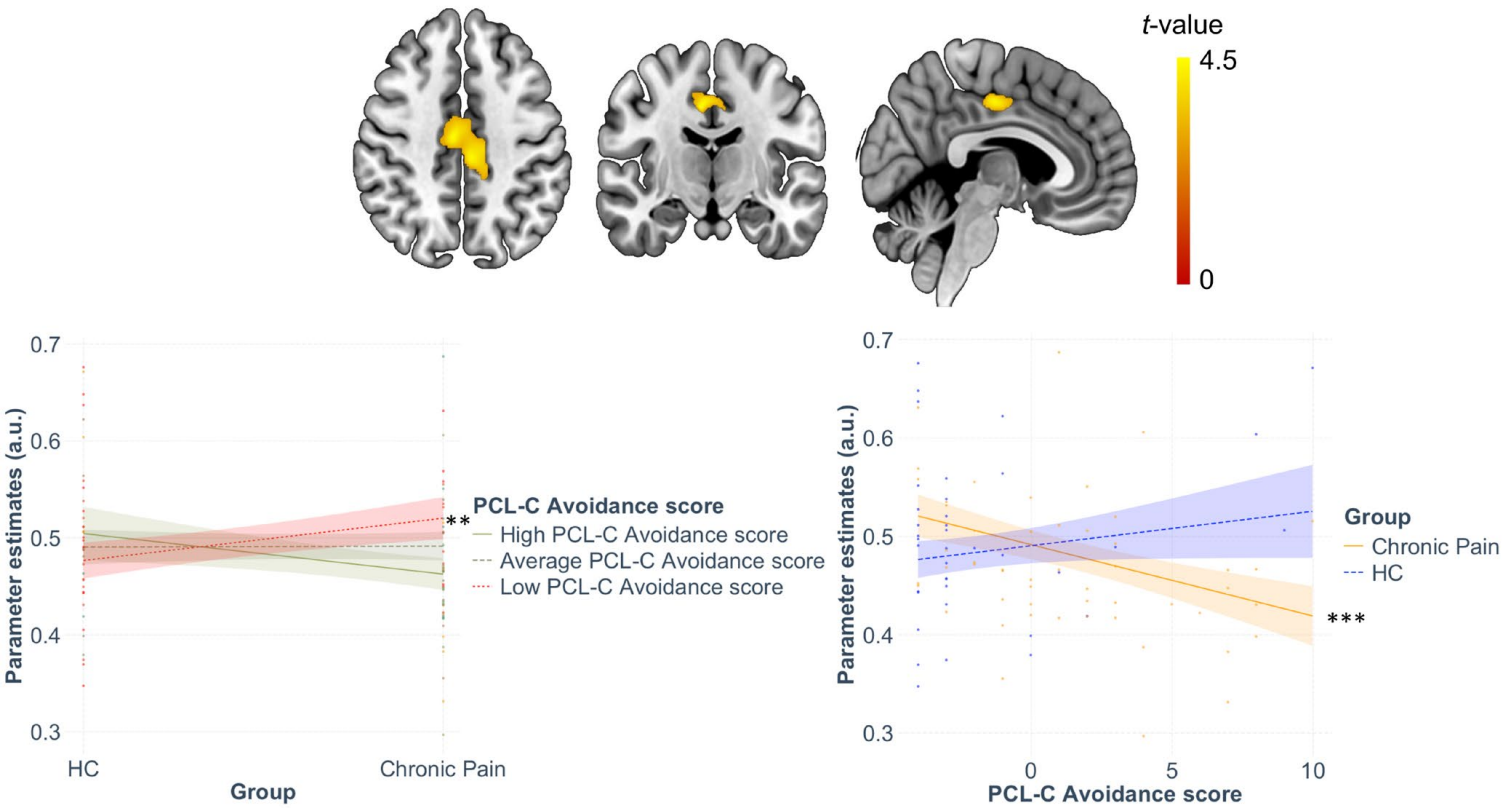
Whole‐brain association with the group‐by‐avoidance interaction. Severity of avoidance moderated the group difference on MCC volume, with the chronic pain group showing larger GMV in this cluster relative to the HC group at low levels of avoidance (left panel; red dashed line). In addition, increasing avoidance severity was associated with smaller GMV in this cluster in people with chronic pain only (right panel; yellow plain line). **p < 0.01; ***p < 0.001. Colour‐bar represents *t*‐statistics. Coloured band around each line represents 95% confidence intervals.

Although the associations between left and right putamen and avoidance scores were significant, the group‐by‐trauma interaction was significantly associated with variations in grey matter volume of the left MCC only (see Figure 2). The first moderation analysis using PCL‐C avoidance scores as moderator showed significantly larger left MCC in people with chronic pain relative to the HC group only at low levels of avoidance (but not at average or high levels). The second moderation analysis using group as the moderator of the relationship between variations in PCL‐C avoidance scores and variations in grey matter volume of the ROIs indicated that smaller left MCC was significantly associated with increasing levels of avoidance in the chronic pain group only. When exploring associations with whole‐brain VBM maps, a similar significant association between the group‐by‐trauma (avoidance scores) and variations in grey matter volume of the bilateral MCC [peak Montreal Neurological Institute (MNI) coordinates (−6, −27, 42), *k* = 1137 voxels, *t*(*83*) = 4.38, *z* = 4.14, *pFWEc* = 0.017; see Figure 3] was evident. After extraction of the raw signal from the cluster peak, the model was statistically significant (adjusted *R* ^2^ = 0.729, *F*(6,83) = 37.027, *p* < 0.001) and the interaction was significantly associated with variations in GMV in this cluster (*b* = −0.011, standard error (*se*) = 0.004, *t* = −2.835, *p* = 0.006, *95*% confidence interval *(CI)* [−0.018, −0.003]). The first moderation analysis testing PCL‐C avoidance as the moderator of associations between groups and GMV revealed significantly larger GMV in this cluster in the chronic pain group relative to the HC group at low (*b* = 0.044, *se* = 0.014, *t* = 3.186, *p* = 0.002, *95*% CI [0.016, 0.071]), but not average (*b* < 0.001, *se* = 0.013, *t* = 0.942, *p* = 0.942, *95*% CI [−0.024, 0.026]) or high levels of PCL‐C avoidance (*b* = −0.042, *se* = 0.024, *t* = −1.730, *p* = 0.087, *95*% CI [−0.090, 0.006]). The second moderation analysis testing group as the moderator of associations between PCL‐C avoidance and GMV revealed that increasing PCL‐C avoidance scores were significantly associated with decreasing GMV in this cluster in the chronic pain (*b* = −0.007, *se* = 0.002, *t scores* = −3.760, *p* < 0.001, *95*% CI [−0.011, −0.003]) but not the HC group (*b* = 0.004, *se* = 0.003, *t* = 1.080, *p* = 0.283, *95*% CI [−0.003, 0.010]).

##### 3.1.3.3 Hyperarousal

There was no significant effect of group, hyperarousal scores, or group‐by‐trauma interaction for any of the ROIs or with the whole‐brain VBM maps.

## 4 Discussion

This study indicates that the severity of stress symptoms impacts brain morphology differently in people with chronic pain, compared to pain‐free healthy controls. At higher levels of stress symptoms, larger putamen and right posterior insula volumes were evident in HCs compared to chronic pain, while people with chronic pain had smaller left MCC than HCs at lower levels of stress symptoms. Increasing stress severity was associated with larger putamen volumes in HCs, and with smaller left MCC and right posterior insula in people with chronic pain. Additionally, people with chronic pain exhibited smaller right MFG than HCs, independent of stress symptoms severity, and stress symptoms severity was associated with a larger left anterior insula, independent of the group. Exploratory symptom‐specific effects were evident in the putamen and right posterior insula for re‐experiencing symptoms, and in the left MCC for avoidance symptoms.

Consistent with previous studies of various chronic pain conditions (Ong et al. 2019), the right MFG was smaller in people with chronic pain compared to HCs. This key part of the dorsolateral prefrontal cortex is critical for executive functions, and for the control and regulation of emotional expression (Nejati et al. 2021). Decreased dorsolateral prefrontal cortex volume may reflect a loss of dendrites (Kang et al. 2019), neurons, or lack of neurogenesis in this region that may impact these functions (Seminowicz and Moayedi 2017). Our findings could also reflect atypical brain aging in people with chronic pain (Apkarian et al. 2004), which could be a consequence associated with living with chronic pain. However, this interpretation is speculative given this study did not record indices of cognition, brain function, or aging. Other cellular and/or molecular mechanisms not recorded in this study, such as unbalanced neuroendocrine or immune systems, may also contribute to grey matter alterations in this region, as seen in other conditions such as major depressive disorder (Opel et al. 2019). Long‐term consequences of trauma/stress exposure include blunted cortisol levels (Lupien et al. 2009). Blunted levels of cortisol will reduce its inhibitory control on the immune system, leading to chronic elevated low‐grade systemic inflammation (Elenkov and Chrousos 2002). Prolonged exposure to elevated low‐grade inflammation can in turn damage the brain (Marsland et al. 2015), weakening the glucocorticoid negative feedback loops and associated oxidative stress systems. Therefore, future studies are warranted, particularly to investigate the relationship between chronic pain and stress symptoms on cognitive and emotional function in relation to these morphological brain changes.

The severity of stress symptoms was associated with larger left, but not right, anterior insula and was not associated with grey matter volume alterations in other expected stress‐sensitive regions including the hippocampus, amygdala, mPFC, ACC, and posterior insula. This result extends to chronic pain, observations of larger insular volume as a marker of resilience following trauma exposure and associated with trauma severity (Roeckner et al. 2021). However, it is important to note that most previous studies have not investigated the anterior and posterior insulae separately, potentially hindering the identification of more subtle and specific effects of trauma or chronic pain on more spatially refined subregions. Along with the dorsal ACC, the anterior insula is a core region of the salience network (Uddin 2015) that integrates multi‐sensory, exteroceptive and interoceptive stimuli received by the posterior insula (Craig 2005, 2009). Larger anterior insula may thus reflect increased neurogenesis following stress exposure, potentially contributing to the development of better coping strategies with stress. This interpretation will need to be confirmed in future studies.

The severity of stress symptoms moderated the volumetric differences between people with chronic pain and HCs in the bilateral putamen, right posterior insula, and left MCC. Larger putamen volume was associated with more severe stress symptoms, especially re‐experiencing symptoms, in HCs but not in people with chronic pain. There was no overall group difference in these regions, independently of stress severity, and no overall association with stress severity, independently of group. The putamen is a core dopaminergic hub of the dorsal striatum involved in locomotion and supports learning processes (Hardwick et al. 2013). Consistent with a previous study across groups of people exposed (with and without PTSD) and not exposed to trauma, a larger putamen may be related to mechanisms of resilience to stress/trauma in HCs (Zilcha‐Mano et al. 2022). Larger posterior insula and MCC volumes were evident in people with chronic pain relative to HCs at lower levels of stress symptoms, potentially reflecting maladaptive compensatory or recovery effects as a result of developing chronic pain. Moreover, stress severity was associated with smaller volumes of these regions in the chronic pain group. In addition to its role in sensorimotor processing (Craig 2005, 2009), the posterior insula is critical for pain perception (Uddin et al. 2017). Smaller posterior insula may reflect the detrimental effect of chronic stress exposure on the integrity of this region in chronic pain, likely due to chronic exposure to pain, and potentially contributing to aberrant sensorimotor processing (Fleming et al. 2024). The MCC is critically involved in higher cognitive processes (e.g., cognitive control, conflict‐monitoring), body orientation, and movement execution (Vogt 2005), and is key for pain empathy (Bruneau et al. 2015). Consistent with similar findings observed in mental health conditions, including PTSD and psychotic disorders (Quidé et al. 2018; Stevens and Jovanovic 2019), smaller MCC may reflect stress‐related effects on the integrity of larger social cognitive networks in people with chronic pain. This will need to be directly tested in behavioural and functional MRI studies in this population. In the present study, both the ROI and whole‐brain analyses reported specific associations between smaller MCC and avoidance symptoms, consistent with other PTSD studies (Hinojosa et al. 2019) and the role of the MCC in pain (Vogt 2016).

Only brain regions commonly altered in chronic pain and trauma studies were investigated here, and not all the pain matrix was included. However, results suggest that both regions from the lateral (sensory‐discriminative) and medial (affective–motivational processing) pain systems are sensitive to stress/PTSS in people with chronic pain (Pricope et al. 2022). This may have implications on treatment choices: people with chronic pain reporting more severe stress/PTSS symptoms may benefit from interventions targeting both systems, such as combinations of pharmacological (e.g., opioids for action on the medial system) (Zubieta et al. 2001) and neuromodulation approaches (e.g., repetitive transcranial magnetic stimulations on the lateral system) (Gatzinsky et al. 2021). Using this type of combined approach may help modulate more efficiently the descending pain modulatory system (made of the ACC, hypothalamus, amygdalae, parabrachial nuclei, rostral ventromedial medulla, midbrain periaqueductal grey, median raphe nuclei, and locus coeruleus) to better reduce pain experience.

This study presents several limitations. First, the sample size was relatively small, which may partly have hindered the discovery of subtle effects. Second, the chronic pain group included a range of different pain conditions and pain was experienced in different body locations (face, lower body), potentially mitigating condition/location‐specific effects. However, this approach was the most appropriate to identify large and common brain morphology alterations associated with a shared environmental risk factor (stress) and their interactions on brain morphology across various chronic pain conditions. Replication in larger samples, such as in the Enhancing NeuroImaging Genetics through Meta‐Analyses (ENIGMA)‐Chronic Pain working group (Quidé et al. 2024) is needed to confirm and refine these findings. Although different chronic pain conditions were included in this study, the selection was not diverse and does not represent common pain etiologies, limiting the interpretation of the results to the conditions included. Third, ‘scan pain’ intensity was measured following the scanning session and may have been underestimated. To better estimate pain intensity during scanning, future studies may ask the participants to estimate the experienced pain intensity at several stages of the scanning session. Of note, pain intensity either averaged across the week before or during the scanning session was not associated with changes in brain morphology (see Supporting Information Tables S4–S6). We also acknowledge that the neuropathic pain scale was used for participants with spinal cord injury only, hampering a detailed characterisation of patients with neuropathic facial pain. Fourth, most participants with chronic pain used a variety of pharmacological treatments (see Supporting Information Table S1) and it is important to note that medication use/dosage was not considered in these analyses pertaining to the presence of pain‐free, drug‐naïve HCs. Although there was no significant difference in brain morphology between people with chronic pain using medication and those not using pain‐related medications (see Supporting Information Table S7), pharmacological treatments are known to impact brain neurochemistry, morphology, and function (Harris et al. 2013; Lee et al. 2014; Murray et al. 2021), and represent potential confounds that should not be completely ruled out. In addition, participants included in the present study were mostly responsive to some treatment, as suggested by the relatively low levels of pain intensity reported. Finally, this study was cross‐sectional in design and results represent only a static snapshot of the brain state at the time of assessment. As chronic pain conditions are dynamic, especially in response (or at least partially) to medication, future studies may account for these dynamic changes (e.g., pain intensity, phases) using longitudinal designs.

In conclusion, the present study provides new evidence for the role of stress severity in alterations of brain morphology in people with chronic pain. Higher levels of stress were associated with larger putamen and right posterior insula in healthy, chronic pain‐free participants, potentially reflecting mechanisms of resilience to stress in this group. Higher levels of avoidance were associated with smaller left MCC, a core region of the “pain matrix” with strong links to the somatosensory network and critical for empathy, especially toward pain‐related stimuli. This finding is consistent with studies investigating similar stress and trauma‐related brain regions across other psychiatric conditions. Introducing stress‐related interventions that can influence these brain regions and networks (Quidé et al. 2012), either before or in conjunction with pain‐related treatments, may be beneficial to people with chronic pain who report elevated stress symptoms (Lumley et al. 2022). Replication in larger samples is necessary, especially to confirm the specificity of avoidance symptoms on the MCC.

## Author Contributions

Y.Q. contributed conceptualisation, data curation, formal analysis, investigation, methodology, validation, visualisation, writing of the original draft, and review and editing. N.H.‐S. contributed conceptualisation, methodology, and review and editing. N.N.‐N. contributed conceptualisation, methodology, and review and editing. J.H.M. contributed conceptualisation, methodology, supervision, and review and editing. S.M.G. contributed conceptualisation, data curation, formal analysis, funding acquisition, investigation, methodology, project administration, resources, supervision, and review and editing.

## Conflicts of Interest

The authors declare no conflicts of interest.

## Supporting information


Data S1.

## Data Availability

The data that support the findings of this study are available from the corresponding author upon reasonable request.
