# Supplementary material for: Stress‐Related Brain Alterations in Chronic Pain
Source: Eur J Pain. 2025 May 9;29(6):e70034. doi: 10.1002/ejp.70034 (PMC12063716; doi:10.1002/ejp.70034)
Supplement: Supplementary file 1 — Data S1. [file EJP-29-0-s001.docx]

**Stress-related brain alterations in chronic pain**

**- Supplementary Material -**

Yann Quidé^1,2^, Negin Hesam-Shariati^1,2^, Nell Norman-Nott^1,2^, James H. McAuley^2,3^, Sylvia M. Gustin^1,2^

^1^ NeuroRecovery Research Hub, School of Psychology, UNSW Sydney, NSW, Australia

^2^ Centre for Pain IMPACT, Neuroscience Research Australia, Randwick, NSW, Australia

^3^ School of Health Sciences, Faculty of Medicine and Health, UNSW Sydney, NSW, Australia

***Corresponding author:**

Dr Yann Quidé, NeuroRecovery Research Hub, School of Psychology, Biological Sciences (Biolink) building, Level 1, UNSW Sydney, NSW, 2052, Australia.

Tel: +61 2 9065 1883

Email: [y.quide@unsw.edu.au; yannquide@gmail.com](mailto:stephen.wood@orygen.org.au)

Table of Contents

[Table S1. Pain-related medication use details for included people with chronic pain 3](#_Toc193130507)

[Table S2. Results of the moderation analyses for all ROIs including only people with neuropathic pain and healthy controls 4](#_Toc193130508)

[Table S3. Results of the moderation analyses for all ROIs including only people with non-neuropathic pain and healthy controls 5](#_Toc193130509)

[Table S4. Partial correlations between pain severity (pain diary and scan pain) with brain morphology for the whole group of people with chronic pain, as well as within people with neuropathic or non-neuropathic pain 6](#_Toc193130510)

[Table S5. Results of the analyses using scores to the pain diary for all ROIs including (A) all people with chronic pain, and (B) only people with neuropathic pain 7](#_Toc193130511)

[Table S6. Results of the analyses using scores to the scan pain for all ROIs including (A) all people with chronic pain, and (B) only people with neuropathic pain 9](#_Toc193130512)

[Table S7. Results of the analyses comparing people with chronic pain using pain-related medication to people with chronic pain not using pain-related medication 11](#_Toc193130513)

| **Table S1.** Pain-related medication use details for included people with chronic pain |  |
| --- | --- |
| Medication type | N |
| None | 22 |
| NSAID | 1 |
| Antidepressants | 2 |
| Anticonvulsants | 10 |
| NSAID+Acetomiphen | 1 |
| NSAID+antidepressants | 1 |
| NSAID+anticonvulsants | 2 |
| Acetominophen+opioids | 3 |
| Acetominophen+antidepressants | 2 |
| Acetominophen+anticonvulsants | 2 |
| Opioid+antidepressants | 1 |
| Antidepressant+anticonvulsants | 1 |
| 3 or more pain-related medication | 4 |
|  |  |

| **Table S2.** Results of the moderation analyses for all ROIs including only people with neuropathic pain and healthy controls | | | | | | | | | | | | | | | | | | | | | | | |
| --- | --- | --- | --- | --- | --- | --- | --- | --- | --- | --- | --- | --- | --- | --- | --- | --- | --- | --- | --- | --- | --- | --- | --- |
| ROI | Model | | | | Group (Neuropathic pain vs Controls) | | | | | | Stress (PCL-C total score) | | | | | | Group x Stress | | | | | |  |
|  | *Adj R^2^* | *F* | *df* | *p*-value | *b* | *se* | LLCI | ULCI | *t*-value | *p*-value | *b* | *se* | LLCI | ULCI | *t*-value | *p*-value | *b* | *se* | LLCI | ULCI | *t*-value | *p*-value | |
| LNAcc | 0.563 | 12.415 | 6,68 | **<1.92×10−3** | -0.0099 | 0.0102 | -0.0302 | 0.0104 | -0.9713 | 0.3348 | 0.0005 | 0.0011 | -0.0017 | 0.0027 | 0.4420 | 0.6599 | -0.0013 | 0.0014 | -0.0040 | 0.0014 | -0.9404 | 0.3503 | |
| RNAcc | 0.523 | 10.531 | 6,68 | **<1.92×10−3** | -0.0032 | 0.0086 | -0.0204 | 0.0140 | -0.3707 | 0.7120 | 0.0002 | 0.0010 | -0.0017 | 0.0021 | 0.2301 | 0.8187 | -0.0010 | 0.0012 | -0.0033 | 0.0014 | -0.8357 | 0.4063 | |
| LCaud | 0.553 | 15.652 | 6,68 | **<1.92×10−3** | -0.0190 | 0.0720 | -0.1628 | 0.1248 | -0.2636 | 0.7929 | 0.0068 | 0.0067 | -0.0066 | 0.0203 | 1.0150 | 0.3137 | -0.0024 | 0.0084 | -0.0191 | 0.0144 | -0.2803 | 0.7801 | |
| RCaud | 0.475 | 10.400 | 6,68 | **<1.92×10−3** | -0.0279 | 0.0816 | -0.1908 | 0.1350 | -0.3422 | 0.7333 | 0.0038 | 0.0073 | -0.0107 | 0.0184 | 0.5265 | 0.6002 | -0.0015 | 0.0096 | -0.0206 | 0.0176 | -0.1569 | 0.8758 | |
| LPut | 0.539 | 17.126 | 6,68 | **<1.92×10−3** | -0.0402 | 0.0858 | -0.2114 | 0.1310 | -0.4687 | 0.6408 | **0.0241** | **0.0065** | **0.0111** | **0.0371** | **3.6976** | **0.0004** | -0.0181 | 0.0101 | -0.0383 | 0.0021 | -1.7849 | 0.0787 | |
| RPut | 0.541 | 23.214 | 6,68 | **<1.92×10−3** | -0.0490 | 0.0806 | -0.2098 | 0.1119 | -0.6077 | 0.5454 | **0.0217** | **0.0055** | **0.0108** | **0.0326** | **3.9622** | **0.0002** | **-0.0229** | **0.0095** | **-0.0418** | **-0.0040** | **-2.4128** | **0.0185** | |
|  |  |  |  |  |  |  |  |  |  |  |  |  |  |  | **HC** | | **0.0217** | **0.0055** | **0.0108** | **0.0326** | **3.9622** | **0.0002** | |
|  |  |  |  |  |  |  |  |  |  |  |  |  |  |  | Chronic Pain | | -0.0012 | 0.0074 | -0.0159 | 0.0135 | -0.1638 | 0.8704 | |
|  |  |  |  |  |  |  |  |  |  |  |  |  |  |  | Low Trauma | | 0.1574 | 0.1278 | -0.0977 | 0.4125 | 1.2311 | 0.2225 | |
|  |  |  |  |  |  |  |  |  |  |  |  |  |  |  | Average Trauma | | -0.0120 | 0.0848 | -0.1812 | 0.1571 | -0.1419 | 0.8876 | |
|  |  |  |  |  |  |  |  |  |  |  |  |  |  |  | **High Trauma** | | **-0.1814** | **0.0888** | **-0.3586** | **-0.0042** | **-2.0429** | **0.0449** | |
| LPallid | 0.026 | 1.078 | 6.68 | 0.3844 | 0.0052 | 0.0251 | -0.0449 | 0.0554 | 0.2080 | 0.8359 | 0.0005 | 0.0016 | -0.0026 | 0.0037 | 0.3271 | 0.7446 | 0.0009 | 0.0031 | -0.0054 | 0.0072 | 0.2832 | 0.7779 | |
| RPallid | 0.070 | 1.394 | 6,68 | 0.2298 | 0.0118 | 0.0249 | -0.0379 | 0.0615 | 0.4747 | 0.6365 | -0.0003 | 0.0015 | -0.0033 | 0.0027 | -0.1916 | 0.8486 | 0.0019 | 0.0032 | -0.0045 | 0.0083 | 0.6020 | 0.5492 | |
| LAmyg | 0.647 | 25.391 | 6,68 | **<1.92×10−3** | -0.0147 | 0.0168 | -0.0482 | 0.0188 | -0.8773 | 0.3834 | 0.0013 | 0.0017 | -0.0021 | 0.0046 | 0.7637 | 0.4477 | -0.0029 | 0.0022 | -0.0074 | 0.0016 | -1.2879 | 0.2022 | |
| RAmyg | 0.717 | 32.087 | 6,68 | **<1.92×10−3** | -0.0032 | 0.0161 | -0.0354 | 0.0289 | -0.2009 | 0.8414 | 0.0002 | 0.0017 | -0.0032 | 0.0036 | 0.1353 | 0.8928 | -0.0019 | 0.0023 | -0.0065 | 0.0028 | -0.8030 | 0.4248 | |
| LHippo | 0.582 | 14.834 | 6,68 | **<1.92×10−3** | -0.0547 | 0.0691 | -0.1926 | 0.0832 | -0.7912 | 0.4316 | 0.0024 | 0.0070 | -0.0116 | 0.0164 | 0.3422 | 0.7333 | 0.0006 | 0.0095 | -0.0184 | 0.0196 | 0.0661 | 0.9475 | |
| RHippo | 0.601 | 17.918 | 6,68 | **<1.92×10−3** | -0.0261 | 0.0700 | -0.1659 | 0.1137 | -0.3726 | 0.7106 | 0.0012 | 0.0080 | -0.0147 | 0.0171 | 0.1534 | 0.8785 | 0.0023 | 0.0105 | -0.0188 | 0.0233 | 0.2166 | 0.8292 | |
| LThal | 0.144 | 3.817 | 6,68 | 0.0025 | 0.0727 | 0.1306 | -0.1879 | 0.3334 | 0.5569 | 0.5794 | -0.0078 | 0.0157 | -0.0392 | 0.0236 | -0.4963 | 0.6213 | 0.0042 | 0.0192 | -0.0342 | 0.0426 | 0.2195 | 0.8269 | |
| RThal | 0.125 | 3.617 | 6,68 | 0.0036 | 0.2259 | 0.1525 | -0.0784 | 0.5303 | 1.4812 | 0.1432 | -0.0090 | 0.0163 | -0.0415 | 0.0234 | -0.5554 | 0.5805 | 0.0084 | 0.0189 | -0.0292 | 0.0460 | 0.4458 | 0.6571 | |
| LMFC | 0.603 | 23.010 | 6,68 | **<1.92×10−3** | -0.0049 | 0.0528 | -0.1103 | 0.1005 | -0.0930 | 0.9262 | -0.0010 | 0.0057 | -0.0124 | 0.0105 | -0.1695 | 0.8659 | 0.0023 | 0.0072 | -0.0122 | 0.0167 | 0.3149 | 0.7538 | |
| RMFC | 0.652 | 32.651 | 6,68 | **<1.92×10−3** | -0.0477 | 0.0533 | -0.1541 | 0.0587 | -0.8944 | 0.3742 | -0.0009 | 0.0059 | -0.0127 | 0.0108 | -0.1553 | 0.8770 | -0.0009 | 0.0059 | -0.0127 | 0.0108 | -0.1553 | 0.8770 | |
| LAntCgG | 0.676 | 22.738 | 6,68 | **<1.92×10−3** | -0.2126 | 0.1152 | -0.4424 | 0.0173 | -1.8451 | 0.0694 | 0.0087 | 0.0121 | -0.0154 | 0.0328 | 0.7210 | 0.4734 | -0.0115 | 0.0160 | -0.0434 | 0.0204 | -0.7200 | 0.4740 | |
| RAntCgG | 0.547 | 12.008 | 6,68 | **<1.92×10−3** | -0.0068 | 0.1283 | -0.2629 | 0.2493 | -0.0533 | 0.9577 | 0.0114 | 0.0157 | -0.0198 | 0.0427 | 0.7298 | 0.4680 | -0.0175 | 0.0181 | -0.0536 | 0.0185 | -0.9709 | 0.3350 | |
| LMCgG | 0.676 | 24.896 | 6,68 | **<1.92×10−3** | 0.0423 | 0.0870 | -0.1314 | 0.2160 | 0.4860 | 0.6285 | 0.0061 | 0.0094 | -0.0126 | 0.0248 | 0.6532 | 0.5158 | -0.0226 | 0.0125 | -0.0477 | 0.0024 | -1.8057 | 0.0754 | |
| RMCgG | 0.651 | 27.952 | 6,68 | **<1.92×10−3** | 0.0678 | 0.1209 | -0.1734 | 0.3090 | 0.5609 | 0.5767 | 0.0047 | 0.0150 | -0.0252 | 0.0347 | 0.3152 | 0.7536 | -0.0140 | 0.0163 | -0.0466 | 0.0186 | -0.8545 | 0.3958 | |
| LMFC | 0.603 | 23.010 | 6,68 | **<1.92×10−3** | -0.0049 | 0.0528 | -0.1103 | 0.1005 | -0.0930 | 0.9262 | -0.0010 | 0.0057 | -0.0124 | 0.0105 | -0.1695 | 0.8659 | 0.0023 | 0.0072 | -0.0122 | 0.0167 | 0.3149 | 0.7538 | |
| RMFG | 0.652 | 32.651 | 6,68 | **<1.92×10−3** | -0.0477 | 0.0533 | -0.1541 | 0.0587 | -0.8944 | 0.3742 | -0.0009 | 0.0059 | -0.0127 | 0.0108 | -0.1553 | 0.8770 | 0.0040 | 0.0074 | -0.0107 | 0.0188 | 0.5462 | 0.5867 | |
| LAIns | 0.633 | 11.632 | 6,68 | **<1.92×10−3** | -0.0556 | 0.0780 | -0.2112 | 0.1000 | -0.7130 | 0.4783 | **0.0162** | **0.0078** | **0.0007** | **0.0316** | **2.0868** | **0.0407** | -0.0125 | 0.0120 | -0.0363 | 0.0114 | -1.0444 | 0.3000 | |
| RAIns | 0.596 | 11.642 | 6,68 | **<1.92×10−3** | 0.0060 | 0.0716 | -0.1368 | 0.1488 | 0.0845 | 0.9329 | 0.0113 | 0.0064 | -0.0015 | 0.0242 | 1.7630 | 0.0824 | -0.0120 | 0.0096 | -0.0310 | 0.0071 | -1.2536 | 0.2143 | |
| LPIns | 0.679 | 20.413 | 6,68 | **<1.92×10−3** | 0.0530 | 0.0398 | -0.0264 | 0.1323 | 1.3327 | 0.1871 | 0.0043 | 0.0042 | -0.0040 | 0.0127 | 1.0294 | 0.3069 | -0.0083 | 0.0060 | -0.0202 | 0.0036 | -1.3974 | 0.1668 | |
| RPIns | 0.694 | 27.591 | 6,68 | **<1.92×10−3** | 0.0332 | 0.0418 | -0.0503 | 0.1167 | 0.7925 | 0.4308 | 0.0059 | 0.0049 | -0.0038 | 0.0156 | 1.2166 | 0.2280 | -0.0100 | 0.0059 | -0.0219 | 0.0018 | -1.6874 | 0.0961 | |
| ROI: region of interest; PCLC: posttraumatic stress disorder checklist - civilian; L/R: left/right; NAcc: nucleus accumbens; Caud: caudate nucleus; Put: putamen; Pallid: pallidum; Amyg: amygdala; Hippo: hippocampus; Thal: thalamus proper; MFC: medial frontal cortex; AntCgG: anterior cingulate gyrus; MCgG: middle cingulate gyrus; MFG: middle frontal gyrus; AIns: anterior insula; Pins: posterior insula; Adj R^2^: adjusted coefficient of determination; se: standard error; LLCI: bootstrapped 95% lower levels confidence interval; ULCI: bootstrapped 95% upper levels confidence interval  Statistically significant associations (*p*<0.05 within each model) are in bold and highlighted in grey | | | | | | | | | | | | | | | | | | | | | | | |

| **Table S3.** Results of the moderation analyses for all ROIs including only people with non-neuropathic pain and healthy controls | | | | | | | | | | | | | | | | | | | | | | | |
| --- | --- | --- | --- | --- | --- | --- | --- | --- | --- | --- | --- | --- | --- | --- | --- | --- | --- | --- | --- | --- | --- | --- | --- |
| ROI | Model | | | | Group (Non-neuropathic pain vs Controls) | | | | | | Stress (PCL-C total score) | | | | | | Group x Stress | | | | | |  |
|  | *Adj R^2^* | *F* | *df* | *p*-value | *b* | *se* | LLCI | ULCI | *t*-value | *p*-value | *b* | *se* | LLCI | ULCI | *t*-value | *p*-value | *b* | *se* | LLCI | ULCI | *t*-value | *p*-value | |
| LNAcc | 0.657 | 17.816 | 6,46 | **<1.92×10−3** | -0.0244 | 0.0139 | -0.0525 | 0.0036 | -1.7514 | 0.0865 | 0.0003 | 0.0010 | -0.0017 | 0.0022 | 0.2833 | 0.7782 | -0.0002 | 0.0013 | -0.0029 | 0.0024 | -0.1833 | 0.8553 | |
| RNAcc | 0.738 | 35.176 | 6,46 | **<1.92×10−3** | -0.0097 | 0.0096 | -0.0291 | 0.0096 | -1.0114 | 0.3171 | 0.0000 | 0.0007 | -0.0015 | 0.0014 | -0.0225 | 0.9821 | -0.0005 | 0.0009 | -0.0023 | 0.0013 | -0.6073 | 0.5466 | |
| LCaud | 0.622 | 17.065 | 6,46 | **<1.92×10−3** | -0.1260 | 0.0925 | -0.3122 | 0.0603 | -1.3616 | 0.1800 | 0.0060 | 0.0071 | -0.0083 | 0.0202 | 0.8440 | 0.4030 | -0.0035 | 0.0090 | -0.0217 | 0.0147 | -0.3869 | 0.7006 | |
| RCaud | 0.622 | 15.103 | 6,46 | **<1.92×10−3** | -0.1099 | 0.1078 | -0.3269 | 0.1070 | -1.0199 | 0.3131 | 0.0028 | 0.0074 | -0.0122 | 0.0178 | 0.3789 | 0.7065 | -0.0012 | 0.0090 | -0.0194 | 0.0169 | -0.1350 | 0.8932 | |
| LPut | 0.581 | 17.174 | 6,46 | **<1.92×10−3** | -0.2108 | 0.1685 | -0.5499 | 0.1283 | -1.2514 | 0.2171 | **0.0250** | **0.0070** | **0.0109** | **0.0392** | **3.5619** | **0.0009** | **-0.0282** | **0.0127** | **-0.0539** | **-0.0026** | **-2.2132** | **0.0319** | |
|  |  |  |  |  |  |  |  |  |  |  |  |  |  |  | **HC** | | **0.0250** | **0.0070** | **0.0109** | **0.0392** | **3.5619** | **0.0009** | |
|  |  |  |  |  |  |  |  |  |  |  |  |  |  |  | Chronic Pain | | -0.0032 | 0.0107 | -0.0246 | 0.0183 | -0.2973 | 0.7675 | |
|  |  |  |  |  |  |  |  |  |  |  |  |  |  |  | Low Trauma | | 0.0711 | 0.2555 | -0.4432 | 0.5854 | 0.2782 | 0.7821 | |
|  |  |  |  |  |  |  |  |  |  |  |  |  |  |  | Average Trauma | | -0.1934 | 0.1724 | -0.5404 | 0.1536 | -1.1217 | 0.2678 | |
|  |  |  |  |  |  |  |  |  |  |  |  |  |  |  | **High Trauma** | | **-0.4578** | **0.1507** | **-0.7612** | **-0.1545** | **-3.0380** | **0.0039** | |
| RPut | 0.622 | 24.808 | 6,46 | **<1.92×10−3** | -0.2079 | 0.1608 | -0.5317 | 0.1158 | -1.2928 | 0.2025 | **0.0218** | **0.0057** | **0.0104** | **0.0332** | **3.8438** | **0.0004** | -0.0206 | 0.0120 | -0.0449 | 0.0036 | -1.7135 | 0.0934 | |
| LPallid | -0.015 | 0.819 | 6,46 | 0.5610 | -0.0471 | 0.0316 | -0.1108 | 0.0165 | -1.4913 | 0.1427 | 0.0011 | 0.0016 | -0.0022 | 0.0043 | 0.6501 | 0.5189 | 0.0010 | 0.0025 | -0.0041 | 0.0060 | 0.3783 | 0.7069 | |
| RPallid | -0.020 | 1.026 | 6,46 | 0.4205 | -0.0555 | 0.0290 | -0.1140 | 0.0029 | -1.9118 | 0.0621 | 0.0003 | 0.0016 | -0.0030 | 0.0036 | 0.1707 | 0.8652 | 0.0021 | 0.0025 | -0.0029 | 0.0070 | 0.8372 | 0.4068 | |
| LAmyg | 0.788 | 39.847 | 6,46 | **<1.92×10−3** | -0.0053 | 0.0199 | -0.0453 | 0.0348 | -0.2645 | 0.7926 | 0.0009 | 0.0013 | -0.0016 | 0.0035 | 0.7488 | 0.4578 | -0.0032 | 0.0018 | -0.0070 | 0.0005 | -1.7649 | 0.0842 | |
| RAmyg | 0.835 | 41.898 | 6,46 | **<1.92×10−3** | 0.0115 | 0.0211 | -0.0311 | 0.0540 | 0.5436 | 0.5893 | -0.0002 | 0.0014 | -0.0030 | 0.0027 | -0.1127 | 0.9108 | -0.0013 | 0.0018 | -0.0049 | 0.0024 | -0.6945 | 0.4909 | |
| LHippo | 0.638 | 13.204 | 6,46 | **<1.92×10−3** | -0.1079 | 0.0814 | -0.2716 | 0.0559 | -1.3259 | 0.1914 | 0.0009 | 0.0060 | -0.0111 | 0.0129 | 0.1481 | 0.8829 | 0.0064 | 0.0084 | -0.0105 | 0.0233 | 0.7663 | 0.4474 | |
| RHippo | 0.670 | 19.897 | 6,46 | **<1.92×10−3** | -0.0432 | 0.0818 | -0.2079 | 0.1215 | -0.5283 | 0.5998 | -0.0003 | 0.0067 | -0.0137 | 0.0132 | -0.0429 | 0.9659 | 0.0073 | 0.0095 | -0.0118 | 0.0263 | 0.7680 | 0.4464 | |
| LThal | 0.181 | 2.234 | 6,46 | 0.0565 | 0.0920 | 0.2537 | -0.4188 | 0.6027 | 0.3625 | 0.7186 | -0.0081 | 0.0148 | -0.0378 | 0.0216 | -0.5476 | 0.5866 | 0.0051 | 0.0226 | -0.0404 | 0.0507 | 0.2275 | 0.8211 | |
| RThal | 0.277 | 4.564 | 6,46 | **<1.92×10−3** | 0.1853 | 0.2486 | -0.3151 | 0.6856 | 0.7454 | 0.4598 | -0.0100 | 0.0149 | -0.0399 | 0.0199 | -0.6722 | 0.5048 | 0.0110 | 0.0221 | -0.0334 | 0.0554 | 0.4971 | 0.6215 | |
| LMFC | 0.705 | 15.918 | 6,46 | **<1.92×10−3** | -0.0918 | 0.0704 | -0.2336 | 0.0500 | -1.3035 | 0.1989 | -0.0015 | 0.0058 | -0.0132 | 0.0102 | -0.2514 | 0.8026 | 0.0075 | 0.0068 | -0.0061 | 0.0211 | 1.1165 | 0.2700 | |
| RMFC | 0.722 | 28.752 | 6,46 | **<1.92×10−3** | -0.0447 | 0.0725 | -0.1907 | 0.1012 | -0.6169 | 0.5404 | -0.0019 | 0.0063 | -0.0145 | 0.0107 | -0.3082 | 0.7593 | 0.0035 | 0.0073 | -0.0111 | 0.0182 | 0.4854 | 0.6297 | |
| LAntCgG | 0.794 | 40.407 | 6,46 | **<1.92×10−3** | -0.0699 | 0.1426 | -0.3568 | 0.2171 | -0.4901 | 0.6264 | 0.0059 | 0.0090 | -0.0122 | 0.0241 | 0.6579 | 0.5139 | -0.0146 | 0.0110 | -0.0368 | 0.0077 | -1.3182 | 0.1939 | |
| RAntCgG | 0.586 | 10.787 | 6,46 | **<1.92×10−3** | 0.0340 | 0.1602 | -0.2885 | 0.3566 | 0.2124 | 0.8327 | 0.0098 | 0.0147 | -0.0198 | 0.0394 | 0.6657 | 0.5089 | -0.0169 | 0.0158 | -0.0487 | 0.0150 | -1.0670 | 0.2915 | |
| LMCgG | 0.780 | 33.845 | 6,46 | **<1.92×10−3** | -0.0507 | 0.1092 | -0.2705 | 0.1690 | -0.4649 | 0.6442 | 0.0052 | 0.0081 | -0.0111 | 0.0215 | 0.6400 | 0.5254 | -0.0172 | 0.0112 | -0.0397 | 0.0053 | -1.5361 | 0.1314 | |
| RMCgG | 0.715 | 28.132 | 6,46 | **<1.92×10−3** | 0.0649 | 0.1414 | -0.2197 | 0.3494 | 0.4590 | 0.6484 | 0.0044 | 0.0147 | -0.0253 | 0.0340 | 0.2959 | 0.7686 | -0.0202 | 0.0157 | -0.0517 | 0.0113 | -1.2881 | 0.2042 | |
| LMFG | 0.739 | 15.505 | 6,46 | **<1.92×10−3** | -0.5507 | 0.6013 | -1.7610 | 0.6595 | -0.9160 | 0.3645 | 0.0264 | 0.0326 | -0.0393 | 0.0920 | 0.8079 | 0.4233 | -0.0421 | 0.0509 | -0.1446 | 0.0605 | -0.8257 | 0.4132 | |
| RMFG | 0.805 | 31.336 | 6,46 | **<1.92×10−3** | -0.8534 | 0.3805 | -1.6193 | -0.0875 | -2.2430 | 0.0298 | 0.0313 | 0.0201 | -0.0091 | 0.0717 | 1.5597 | 0.1257 | -0.0215 | 0.0292 | -0.0803 | 0.0374 | -0.7335 | 0.4670 | |
| LAIns | 0.765 | 24.125 | 6,46 | **<1.92×10−3** | -0.1194 | 0.0897 | -0.2998 | 0.0611 | -1.3313 | 0.1897 | **0.0155** | **0.0064** | **0.0027** | **0.0283** | **2.4300** | **0.0191** | -0.0111 | 0.0104 | -0.0319 | 0.0098 | -1.0679 | 0.2912 | |
| RAIns | 0.727 | 15.594 | 6,46 | **<1.92×10−3** | -0.0802 | 0.0858 | -0.2529 | 0.0926 | -0.9340 | 0.3552 | 0.0105 | 0.0056 | -0.0008 | 0.0217 | 1.8746 | 0.0672 | -0.0033 | 0.0098 | -0.0230 | 0.0164 | -0.3343 | 0.7397 | |
| LPIns | 0.810 | 28.257 | 6,46 | **<1.92×10−3** | -0.0261 | 0.0429 | -0.1125 | 0.0603 | -0.6078 | 0.5463 | 0.0038 | 0.0038 | -0.0038 | 0.0114 | 1.0027 | 0.3213 | -0.0033 | 0.0045 | -0.0123 | 0.0058 | -0.7308 | 0.4686 | |
| RPIns | 0.745 | 23.683 | 6,46 | **<1.92×10−3** | -0.0419 | 0.0538 | -0.1501 | 0.0664 | -0.7786 | 0.4402 | 0.0055 | 0.0047 | -0.0040 | 0.0150 | 1.1610 | 0.2516 | -0.0101 | 0.0056 | -0.0213 | 0.0011 | -1.8154 | 0.0760 | |
| ROI: region of interest; PCLC: posttraumatic stress disorder checklist - civilian; L/R: left/right; NAcc: nucleus accumbens; Caud: caudate nucleus; Put: putamen; Pallid: pallidum; Amyg: amygdala; Hippo: hippocampus; Thal: thalamus proper; MFC: medial frontal cortex; AntCgG: anterior cingulate gyrus; MCgG: middle cingulate gyrus; MFG: middle frontal gyrus; AIns: anterior insula; Pins: posterior insula; Adj R^2^: adjusted coefficient of determination; se: standard error; LLCI: bootstrapped 95% lower levels confidence interval; ULCI: bootstrapped 95% upper levels confidence interval  Statistically significant associations (*p*<0.05 within each model) are in bold and highlighted in grey | | | | | | | | | | | | | | | | | | | | | | | |

| **Table S4.** Partial correlations between pain severity (pain diary and scan pain) with brain morphology for the whole group of people with chronic pain, as well as within people with neuropathic or non-neuropathic pain | | | | | | | | | | | | |
| --- | --- | --- | --- | --- | --- | --- | --- | --- | --- | --- | --- | --- |
|  | Chronic pain | | | | Neuropathic pain | | | | Non Neuropathic | | | |
|  | pain diary | | scan pain | | pain diary | | scan pain | | pain diary | | scan pain | |
| Region | Correlation | p-value | Correlation | p-value | Correlation | p-value | Correlation | p-value | Correlation | p-value | Correlation | p-value |
| LNAcc | 0.132 | 0.513 | 0.174 | 0.385 | 0.048 | 0.864 | 0.179 | 0.524 | 0.185 | 0.633 | 0.304 | 0.427 |
| RNAcc | 0.114 | 0.572 | 0.105 | 0.604 | 0.183 | 0.514 | 0.199 | 0.477 | -0.158 | 0.685 | 0.017 | 0.966 |
| LAmyg | -0.157 | 0.434 | -0.292 | 0.139 | -0.233 | 0.404 | -0.111 | 0.693 | 0.124 | 0.751 | -0.551 | 0.124 |
| RAmyg | -0.162 | 0.419 | -0.229 | 0.250 | -0.228 | 0.414 | -0.043 | 0.88 | -0.070 | 0.858 | -0.605 | 0.084 |
| LCaud | 0.025 | 0.900 | 0.132 | 0.511 | 0.015 | 0.959 | -0.012 | 0.967 | -0.225 | 0.561 | 0.448 | 0.227 |
| RCaud | -0.048 | 0.812 | 0.036 | 0.860 | -0.076 | 0.787 | -0.036 | 0.898 | -0.328 | 0.389 | 0.266 | 0.490 |
| LHippo | 0.063 | 0.756 | 0.056 | 0.780 | -0.078 | 0.783 | 0.023 | 0.936 | 0.190 | 0.625 | -0.052 | 0.895 |
| RHippo | 0.033 | 0.872 | -0.002 | 0.993 | -0.140 | 0.618 | -0.006 | 0.984 | 0.264 | 0.492 | -0.191 | 0.622 |
| LPallid | 0.351 | 0.073 | 0.242 | 0.224 | 0.449 | 0.094 | 0.251 | 0.367 | 0.258 | 0.502 | 0.549 | 0.126 |
| RPallid | 0.255 | 0.198 | 0.117 | 0.56 | 0.354 | 0.196 | 0.140 | 0.618 | 0.187 | 0.629 | 0.463 | 0.209 |
| LPut | 0.208 | 0.299 | 0.241 | 0.226 | 0.33 | 0.230 | 0.355 | 0.194 | 0.062 | 0.874 | 0.427 | 0.252 |
| RPut | 0.224 | 0.26 | 0.252 | 0.206 | 0.233 | 0.402 | 0.252 | 0.364 | 0.244 | 0.528 | 0.557 | 0.119 |
| LThal | -0.155 | 0.439 | -0.279 | 0.159 | -0.050 | 0.859 | 0.055 | 0.847 | -0.357 | 0.345 | **-0.754** | **0.019** |
| RThal | -0.069 | 0.731 | -0.111 | 0.581 | 0.208 | 0.457 | 0.299 | 0.279 | -0.465 | 0.207 | **-0.712** | **0.031** |
| LAntCgG | -0.213 | 0.287 | -0.334 | 0.088 | -0.456 | 0.088 | -0.338 | 0.218 | 0.270 | 0.482 | -0.369 | 0.328 |
| RAntCgG | 0.169 | 0.400 | -0.099 | 0.623 | -0.040 | 0.887 | 0.122 | 0.665 | 0.401 | 0.285 | -0.330 | 0.386 |
| LAIns | 0.177 | 0.378 | 0.190 | 0.342 | 0.281 | 0.310 | 0.455 | 0.088 | 0.254 | 0.509 | -0.125 | 0.749 |
| RAIns | 0.133 | 0.509 | 0.159 | 0.428 | 0.146 | 0.604 | 0.219 | 0.432 | 0.507 | 0.163 | 0.231 | 0.549 |
| LMCgG | -0.123 | 0.542 | -0.314 | 0.111 | -0.206 | 0.461 | -0.034 | 0.904 | -0.010 | 0.980 | -0.642 | 0.062 |
| RMCgG | 0.055 | 0.785 | -0.174 | 0.384 | -0.051 | 0.856 | 0.050 | 0.860 | 0.282 | 0.463 | -0.411 | 0.271 |
| LMFC | -0.015 | 0.942 | -0.097 | 0.629 | -0.405 | 0.134 | -0.376 | 0.168 | 0.684 | 0.042 | 0.403 | 0.282 |
| RMFC | 0.011 | 0.958 | -0.115 | 0.569 | -0.248 | 0.372 | -0.324 | 0.239 | 0.386 | 0.305 | -0.005 | 0.990 |
| LMFG | 0.098 | 0.626 | -0.140 | 0.485 | -0.224 | 0.421 | -0.153 | 0.587 | 0.613 | 0.079 | -0.125 | 0.750 |
| RMFG | 0.024 | 0.905 | -0.064 | 0.749 | -0.189 | 0.499 | -0.092 | 0.745 | 0.737 | 0.024 | 0.035 | 0.929 |
| LPIns | -0.186 | 0.352 | -0.264 | 0.184 | -0.316 | 0.251 | -0.293 | 0.289 | 0.116 | 0.767 | -0.209 | 0.589 |
| RPIns | -0.273 | 0.168 | -0.329 | 0.094 | -0.444 | 0.098 | -0.382 | 0.160 | -0.117 | 0.765 | -0.334 | 0.379 |
| ROI: region of interest; L/R: left/right; NAcc: nucleus accumbens; Caud: caudate nucleus; Put: putamen; Pallid: pallidum; Amyg: amygdala; Hippo: hippocampus; Thal: thalamus proper; MFC: medial frontal cortex; AntCgG: anterior cingulate gyrus; MCgG: middle cingulate gyrus; MFG: middle frontal gyrus; AIns: anterior insula; Pins: posterior insula Statistically significant associations (p<0.05 within each model) are in bold and highlighted in grey | | | | | | | | | | | | |

| **Table S5.** Results of the analyses using scores to the pain diary for all ROIs including (A) all people with chronic pain, and (B) only people with neuropathic pain | | | | | | | | | | | | | | | | | | | | | | | |
| --- | --- | --- | --- | --- | --- | --- | --- | --- | --- | --- | --- | --- | --- | --- | --- | --- | --- | --- | --- | --- | --- | --- | --- |
| ROI | Model | | | | Pain intensity (Pain diary score) | | | | | | Stress (PCL-C total score) | | | | | | Group x Stress | | | | | |  |
|  | *Adj R^2^* | *F* | *df* | *p*-value | *b* | *se* | LLCI | ULCI | *t*-value | *p*-value | *b* | *se* | LLCI | ULCI | *t*-value | *p*-value | *b* | *se* | LLCI | ULCI | *t*-value | *p*-value | |
| **A. All chronic pain conditions** | | |  |  |  |  |  |  |  |  |  |  |  |  |  |  |  |  |  |  |  |  | |
| LNAcc | **0.586** | **8.551** | 8,26 | **<1.92×10−3** | 0.0013 | 0.0036 | -0.0061 | 0.0086 | 0.3600 | 0.7218 | -0.0010 | 0.0006 | -0.0022 | 0.0003 | -1.6355 | 0.1140 | 0.0001 | 0.0006 | -0.0011 | 0.0012 | 0.1122 | 0.9115 | |
| RNAcc | 0.554 | 8.385 | 8,26 | **<1.92×10−3** | 0.0027 | 0.0043 | -0.0062 | 0.0116 | 0.6307 | 0.5338 | **-0.0014** | **0.0007** | **-0.0028** | **-0.0001** | **-2.1516** | **0.0409** | 0.0000 | 0.0004 | -0.0008 | 0.0007 | -0.0428 | 0.9662 | |
| LCaud | 0.479 | 4.910 | 8,26 | **<1.92×10−3** | -0.0181 | 0.0410 | -0.1023 | 0.0661 | -0.4427 | 0.6617 | 0.0047 | 0.0058 | -0.0071 | 0.0165 | 0.8180 | 0.4208 | 0.0002 | 0.0044 | -0.0088 | 0.0091 | 0.0411 | 0.9676 | |
| RCaud | 0.439 | 6.378 | 8,26 | **<1.92×10−3** | -0.0272 | 0.0425 | -0.1145 | 0.0601 | -0.6405 | 0.5274 | 0.0013 | 0.0064 | -0.0119 | 0.0144 | 0.2008 | 0.8425 | 0.0020 | 0.0058 | -0.0099 | 0.0139 | 0.3472 | 0.7312 | |
| LPut | 0.408 | 3.569 | 8,26 | 0.0064 | 0.0358 | 0.0444 | -0.0555 | 0.1270 | 0.8056 | 0.4278 | -0.0002 | 0.0078 | -0.0162 | 0.0158 | -0.0240 | 0.9810 | -0.0011 | 0.0048 | -0.0110 | 0.0088 | -0.2282 | 0.8213 | |
| RPut | 0.459 | 6.193 | 8,26 | **<1.92×10−3** | 0.0368 | 0.0375 | -0.0403 | 0.1139 | 0.9814 | 0.3354 | -0.0011 | 0.0072 | -0.0159 | 0.0137 | -0.1529 | 0.8796 | -0.0039 | 0.0046 | -0.0134 | 0.0057 | -0.8333 | 0.4123 | |
| LPallid | 0.307 | 2.104 | 8,26 | 0.0726 | 0.0196 | 0.0166 | -0.0145 | 0.0538 | 1.1815 | 0.2481 | -0.0012 | 0.0023 | -0.0060 | 0.0036 | -0.5012 | 0.6205 | -0.0009 | 0.0013 | -0.0037 | 0.0018 | -0.7031 | 0.4883 | |
| RPallid | 0.169 | 1.981 | 8,26 | 0.0898 | 0.0155 | 0.0159 | -0.0171 | 0.0481 | 0.9762 | 0.3380 | -0.0009 | 0.0027 | -0.0064 | 0.0047 | -0.3239 | 0.7486 | -0.0005 | 0.0013 | -0.0032 | 0.0022 | -0.3975 | 0.6942 | |
| LAmyg | 0.646 | 10.885 | 8,26 | **<1.92×10−3** | 0.0047 | 0.0069 | -0.0096 | 0.0189 | 0.6744 | 0.5060 | **-0.0040** | **0.0017** | **-0.0076** | **-0.0004** | **-2.2897** | **0.0304** | 0.0002 | 0.0008 | -0.0014 | 0.0018 | 0.2628 | 0.7947 | |
| RAmyg | 0.608 | 8.183 | 8,26 | **<1.92×10−3** | 0.0019 | 0.0092 | -0.0170 | 0.0209 | 0.2089 | 0.8362 | -0.0030 | 0.0019 | -0.0069 | 0.0010 | -1.5505 | 0.1331 | 0.0002 | 0.0010 | -0.0018 | 0.0021 | 0.1576 | 0.8760 | |
| LHippo | 0.432 | 6.597 | 8,26 | **<1.92×10−3** | 0.0188 | 0.0315 | -0.0459 | 0.0836 | 0.5975 | 0.5553 | -0.0001 | 0.0076 | -0.0158 | 0.0156 | -0.0161 | 0.9873 | 0.0032 | 0.0038 | -0.0047 | 0.0110 | 0.8279 | 0.4152 | |
| RHippo | 0.511 | 7.750 | 8,26 | **<1.92×10−3** | 0.0187 | 0.0350 | -0.0533 | 0.0906 | 0.5331 | 0.5985 | -0.0007 | 0.0085 | -0.0181 | 0.0167 | -0.0819 | 0.9354 | 0.0040 | 0.0039 | -0.0041 | 0.0121 | 1.0208 | 0.3168 | |
| LThal | 0.253 | 3.838 | 8,26 | 0.0042 | -0.0232 | 0.0722 | -0.1716 | 0.1252 | -0.3219 | 0.7501 | -0.0088 | 0.0172 | -0.0442 | 0.0266 | -0.5092 | 0.6149 | 0.0055 | 0.0066 | -0.0082 | 0.0191 | 0.8211 | 0.4191 | |
| RThal | 0.226 | 2.095 | 8,26 | 0.0737 | -0.0047 | 0.0674 | -0.1433 | 0.1338 | -0.0704 | 0.9444 | 0.0002 | 0.0145 | -0.0296 | 0.0301 | 0.0166 | 0.9869 | 0.0009 | 0.0063 | -0.0120 | 0.0138 | 0.1475 | 0.8839 | |
| LMFC | 0.481 | 6.926 | 8,26 | **<1.92×10−3** | 0.0020 | 0.0267 | -0.0530 | 0.0569 | 0.0735 | 0.9420 | 0.0033 | 0.0057 | -0.0083 | 0.0150 | 0.5887 | 0.5612 | -0.0003 | 0.0039 | -0.0083 | 0.0078 | -0.0675 | 0.9467 | |
| RMFC | 0.474 | 3.435 | 8,26 | 0.0079 | -0.0048 | 0.0198 | -0.0454 | 0.0358 | -0.2433 | 0.8097 | 0.0045 | 0.0053 | -0.0063 | 0.0153 | 0.8625 | 0.3963 | -0.0007 | 0.0028 | -0.0064 | 0.0051 | -0.2469 | 0.8069 | |
| LAntCgG | 0.534 | 8.788 | 8,26 | **<1.92×10−3** | -0.0579 | 0.0570 | -0.1751 | 0.0593 | -1.0157 | 0.3192 | -0.0022 | 0.0126 | -0.0280 | 0.0237 | -0.1735 | 0.8636 | -0.0006 | 0.0069 | -0.0148 | 0.0135 | -0.0936 | 0.9262 | |
| RAntCgG | 0.540 | 11.260 | 8,26 | **<1.92×10−3** | 0.0347 | 0.0408 | -0.0492 | 0.1186 | 0.8496 | 0.4033 | -0.0092 | 0.0090 | -0.0278 | 0.0094 | -1.0192 | 0.3175 | -0.0013 | 0.0043 | -0.0102 | 0.0076 | -0.2964 | 0.7693 | |
| LMCgG | 0.619 | 7.158 | 8,26 | **<1.92×10−3** | 0.0042 | 0.0384 | -0.0746 | 0.0831 | 0.1103 | 0.9130 | -0.0146 | 0.0087 | -0.0324 | 0.0032 | -1.6849 | 0.1040 | -0.0001 | 0.0055 | -0.0114 | 0.0113 | -0.0121 | 0.9905 | |
| RMCgG | 0.660 | 11.591 | 8,26 | **<1.92×10−3** | 0.0376 | 0.0377 | -0.0399 | 0.1150 | 0.9969 | 0.3280 | **-0.0170** | **0.0080** | **-0.0335** | **-0.0005** | **-2.1183** | **0.0439** | -0.0004 | 0.0041 | -0.0088 | 0.0079 | -0.1082 | 0.9147 | |
| LMFG | 0.641 | 9.192 | 8,26 | **<1.92×10−3** | 0.0251 | 0.1754 | -0.3355 | 0.3857 | 0.1431 | 0.8873 | -0.0247 | 0.0368 | -0.1003 | 0.0508 | -0.6728 | 0.5070 | -0.0014 | 0.0185 | -0.0395 | 0.0367 | -0.0746 | 0.9411 | |
| RMFG | 0.716 | 18.567 | 8,26 | **<1.92×10−3** | 0.0347 | 0.1903 | -0.3565 | 0.4260 | 0.1825 | 0.8566 | -0.0194 | 0.0247 | -0.0701 | 0.0313 | -0.7864 | 0.4387 | 0.0047 | 0.0237 | -0.0440 | 0.0534 | 0.1991 | 0.8437 | |
| LAIns | 0.433 | 3.338 | 8,26 | 0.0092 | 0.0112 | 0.0343 | -0.0593 | 0.0817 | 0.3268 | 0.7464 | 0.0000 | 0.0076 | -0.0156 | 0.0155 | -0.0058 | 0.9954 | 0.0004 | 0.0042 | -0.0083 | 0.0091 | 0.0928 | 0.9268 | |
| RAIns | 0.488 | 2.982 | 8,26 | 0.0165 | -0.0009 | 0.0341 | -0.0710 | 0.0692 | -0.0254 | 0.9799 | 0.0023 | 0.0078 | -0.0138 | 0.0184 | 0.2908 | 0.7735 | -0.0026 | 0.0036 | -0.0100 | 0.0048 | -0.7324 | 0.4705 | |
| LPIns | 0.678 | 10.845 | 8,26 | **<1.92×10−3** | -0.0141 | 0.0177 | -0.0505 | 0.0223 | -0.7949 | 0.4339 | -0.0022 | 0.0043 | -0.0111 | 0.0066 | -0.5132 | 0.6121 | -0.0006 | 0.0022 | -0.0051 | 0.0038 | -0.2891 | 0.7748 | |
| RPIns | 0.735 | 17.315 | 8,26 | **<1.92×10−3** | -0.0086 | 0.0200 | -0.0496 | 0.0325 | -0.4292 | 0.6713 | -0.0039 | 0.0040 | -0.0120 | 0.0043 | -0.9739 | 0.3391 | 0.0008 | 0.0024 | -0.0042 | 0.0058 | 0.3131 | 0.7567 | |
|  |  |  |  |  |  |  |  |  |  |  |  |  |  |  |  |  |  |  |  |  |  |  | |
| **B. Neuropathic pain only** | | |  |  |  |  |  |  |  |  |  |  |  |  |  |  |  |  |  |  |  |  | |
| LNAcc | 0.615 | 16.660 | 8,14 | **<1.92×10−3** | -0.0016 | 0.0055 | -0.0134 | 0.0103 | -0.2817 | 0.7823 | **-0.0017** | **0.0008** | **-0.0034** | **-0.0001** | **-2.2404** | **0.0418** | 0.0005 | 0.0006 | -0.0007 | 0.0017 | 0.8940 | 0.3865 | |
| RNAcc | 0.431 | 2.603 | 8,14 | 0.0563 | 0.0040 | 0.0055 | -0.0079 | 0.0158 | 0.7161 | 0.4857 | -0.0021 | 0.0013 | -0.0049 | 0.0008 | -1.5342 | 0.1473 | -0.0002 | 0.0007 | -0.0017 | 0.0013 | -0.2830 | 0.7813 | |
| LCaud | 0.372 | 4.088 | 8,14 | 0.0105 | -0.0322 | 0.0557 | -0.1517 | 0.0874 | -0.5774 | 0.5729 | -0.0026 | 0.0115 | -0.0274 | 0.0221 | -0.2271 | 0.8236 | 0.0065 | 0.0087 | -0.0123 | 0.0252 | 0.7385 | 0.4724 | |
| RCaud | 0.326 | 2.427 | 8,14 | 0.0702 | -0.0543 | 0.0595 | -0.1818 | 0.0733 | -0.9121 | 0.3772 | -0.0042 | 0.0136 | -0.0333 | 0.0250 | -0.3052 | 0.7647 | 0.0091 | 0.0112 | -0.0149 | 0.0331 | 0.8149 | 0.4288 | |
| LPut | 0.303 | 2.696 | 8,14 | 0.0502 | 0.0532 | 0.0551 | -0.0650 | 0.1713 | 0.9649 | 0.3510 | 0.0046 | 0.0146 | -0.0267 | 0.0359 | 0.3152 | 0.7572 | 0.0025 | 0.0099 | -0.0188 | 0.0238 | 0.2523 | 0.8045 | |
| RPut | 0.257 | 1.135 | 8,14 | 0.3988 | 0.0474 | 0.0596 | -0.0804 | 0.1752 | 0.7950 | 0.4399 | -0.0027 | 0.0177 | -0.0407 | 0.0353 | -0.1539 | 0.8799 | 0.0014 | 0.0128 | -0.0259 | 0.0288 | 0.1122 | 0.9122 | |
| LPallid | 0.240 | 1.024 | 8,14 | 0.4622 | 0.0231 | 0.0242 | -0.0289 | 0.0750 | 0.9529 | 0.3568 | 0.0012 | 0.0043 | -0.0079 | 0.0104 | 0.2827 | 0.7816 | -0.0006 | 0.0032 | -0.0073 | 0.0062 | -0.1795 | 0.8601 | |
| RPallid | 0.108 | 0.903 | 8,14 | 0.5398 | 0.0162 | 0.0231 | -0.0334 | 0.0657 | 0.7008 | 0.4949 | 0.0014 | 0.0054 | -0.0103 | 0.0130 | 0.2515 | 0.8051 | -0.0003 | 0.0034 | -0.0075 | 0.0069 | -0.0938 | 0.9266 | |
| LAmyg | 0.532 | 5.000 | 8,14 | 0.0044 | 0.0028 | 0.0099 | -0.0183 | 0.0240 | 0.2863 | 0.7788 | **-0.0040** | **0.0018** | **-0.0077** | **-0.0002** | **-2.2782** | **0.0389** | -0.0011 | 0.0010 | -0.0033 | 0.0010 | -1.1388 | 0.2739 | |
| RAmyg | 0.451 | 8.705 | 8,14 | **<1.92×10−3** | -0.0013 | 0.0116 | -0.0261 | 0.0235 | -0.1139 | 0.9110 | -0.0030 | 0.0026 | -0.0087 | 0.0027 | -1.1414 | 0.2728 | -0.0004 | 0.0013 | -0.0032 | 0.0024 | -0.3269 | 0.7486 | |
| LHippo | 0.493 | 6.842 | 8,14 | **<1.92×10−3** | -0.0031 | 0.0417 | -0.0925 | 0.0864 | -0.0736 | 0.9424 | -0.0067 | 0.0130 | -0.0345 | 0.0211 | -0.5145 | 0.6149 | 0.0036 | 0.0062 | -0.0098 | 0.0169 | 0.5732 | 0.5756 | |
| RHippo | 0.510 | 7.044 | 8,14 | **<1.92×10−3** | -0.0178 | 0.0451 | -0.1145 | 0.0790 | -0.3936 | 0.6998 | -0.0052 | 0.0151 | -0.0377 | 0.0273 | -0.3418 | 0.7375 | 0.0057 | 0.0067 | -0.0088 | 0.0201 | 0.8406 | 0.4147 | |
| LThal | 0.074 | 2.596 | 8,14 | 0.0568 | 0.0266 | 0.0929 | -0.1727 | 0.2259 | 0.2864 | 0.7788 | -0.0060 | 0.0201 | -0.0490 | 0.0370 | -0.2982 | 0.7699 | -0.0080 | 0.0095 | -0.0283 | 0.0123 | -0.8443 | 0.4127 | |
| RThal | 0.078 | 1.356 | 8,14 | 0.2951 | 0.1250 | 0.0937 | -0.0759 | 0.3259 | 1.3342 | 0.2035 | -0.0014 | 0.0202 | -0.0448 | 0.0420 | -0.0676 | 0.9470 | -0.0167 | 0.0089 | -0.0358 | 0.0024 | -1.8764 | 0.0816 | |
| LMFC | 0.406 | 3.418 | 8,14 | 0.0215 | -0.0261 | 0.0381 | -0.1077 | 0.0555 | -0.6860 | 0.5039 | 0.0021 | 0.0080 | -0.0150 | 0.0192 | 0.2630 | 0.7964 | 0.0010 | 0.0045 | -0.0087 | 0.0107 | 0.2192 | 0.8297 | |
| RMFC | 0.496 | 3.345 | 8,14 | 0.0234 | -0.0088 | 0.0265 | -0.0656 | 0.0480 | -0.3320 | 0.7448 | 0.0031 | 0.0060 | -0.0097 | 0.0159 | 0.5177 | 0.6128 | -0.0018 | 0.0039 | -0.0102 | 0.0066 | -0.4651 | 0.6490 | |
| LAntCgG | 0.544 | 2.334 | 8,14 | 0.0791 | -0.1155 | 0.0634 | -0.2515 | 0.0205 | -1.8216 | 0.0899 | 0.0051 | 0.0170 | -0.0314 | 0.0415 | 0.2987 | 0.7695 | -0.0067 | 0.0078 | -0.0233 | 0.0100 | -0.8610 | 0.4038 | |
| RAntCgG | 0.475 | 1.658 | 8,14 | 0.1949 | -0.0043 | 0.0643 | -0.1423 | 0.1337 | -0.0669 | 0.9476 | 0.0004 | 0.0127 | -0.0267 | 0.0276 | 0.0346 | 0.9729 | -0.0073 | 0.0075 | -0.0235 | 0.0088 | -0.9712 | 0.3479 | |
| LMCgG | 0.523 | 11.987 | 8,14 | **<1.92×10−3** | -0.0291 | 0.0492 | -0.1347 | 0.0764 | -0.5916 | 0.5635 | -0.0166 | 0.0117 | -0.0416 | 0.0084 | -1.4255 | 0.1759 | 0.0002 | 0.0071 | -0.0150 | 0.0154 | 0.0255 | 0.9800 | |
| RMCgG | 0.568 | 10.310 | 8,14 | **<1.92×10−3** | -0.0174 | 0.0599 | -0.1458 | 0.1110 | -0.2903 | 0.7758 | -0.0157 | 0.0124 | -0.0423 | 0.0109 | -1.2656 | 0.2263 | -0.0003 | 0.0077 | -0.0169 | 0.0163 | -0.0385 | 0.9698 | |
| LMFG | 0.710 | 4.144 | 8,14 | 0.0100 | -0.1396 | 0.1544 | -0.4709 | 0.1916 | -0.9040 | 0.3813 | -0.0395 | 0.0509 | -0.1488 | 0.0697 | -0.7760 | 0.4507 | -0.0014 | 0.0195 | -0.0431 | 0.0404 | -0.0699 | 0.9453 | |
| RMFG | 0.711 | 16.688 | 8,14 | **<1.92×10−3** | -0.1495 | 0.1457 | -0.4620 | 0.1629 | -1.0264 | 0.3221 | -0.0426 | 0.0343 | -0.1161 | 0.0309 | -1.2423 | 0.2345 | 0.0274 | 0.0194 | -0.0142 | 0.0691 | 1.4127 | 0.1796 | |
| LAIns | 0.084 | 1.142 | 8,14 | 0.3954 | 0.0239 | 0.0485 | -0.0801 | 0.1279 | 0.4932 | 0.6295 | -0.0052 | 0.0103 | -0.0273 | 0.0169 | -0.5050 | 0.6214 | 0.0037 | 0.0074 | -0.0121 | 0.0195 | 0.5006 | 0.6244 | |
| RAIns | 0.171 | 2.227 | 8,14 | 0.0909 | 0.0388 | 0.0549 | -0.0788 | 0.1565 | 0.7075 | 0.4909 | -0.0067 | 0.0109 | -0.0300 | 0.0166 | -0.6182 | 0.5463 | -0.0017 | 0.0057 | -0.0138 | 0.0104 | -0.3004 | 0.7683 | |
| LPIns | 0.610 | 6.463 | 8,14 | 0.0013 | -0.0215 | 0.0217 | -0.0681 | 0.0251 | -0.9890 | 0.3394 | -0.0027 | 0.0071 | -0.0179 | 0.0125 | -0.3853 | 0.7058 | -0.0005 | 0.0024 | -0.0056 | 0.0046 | -0.2242 | 0.8258 | |
| RPIns | 0.726 | 7.915 | 8,14 | **<1.92×10−3** | -0.0186 | 0.0184 | -0.0582 | 0.0209 | -1.0103 | 0.3295 | -0.0024 | 0.0057 | -0.0146 | 0.0097 | -0.4301 | 0.6737 | 0.0004 | 0.0026 | -0.0052 | 0.0061 | 0.1662 | 0.8704 | |
| ROI: region of interest; PCLC: posttraumatic stress disorder checklist - civilian; L/R: left/right; NAcc: nucleus accumbens; Caud: caudate nucleus; Put: putamen; Pallid: pallidum; Amyg: amygdala; Hippo: hippocampus; Thal: thalamus proper; MFC: medial frontal cortex; AntCgG: anterior cingulate gyrus; MCgG: middle cingulate gyrus; MFG: middle frontal gyrus; AIns: anterior insula; Pins: posterior insula; Adj R^2^: adjusted coefficient of determination; se: standard error; LLCI: bootstrapped 95% lower levels confidence interval; ULCI: bootstrapped 95% upper levels confidence interval  Statistically significant associations (*p*<0.05 within each model) are in bold and highlighted in grey | | | | | | | | | | | | | | | | | | | | | | | |

| **Table S6.** Results of the analyses using scores to the scan pain for all ROIs including (A) all people with chronic pain, and (B) only people with neuropathic pain | | | | | | | | | | | | | | | | | | | | | | | |
| --- | --- | --- | --- | --- | --- | --- | --- | --- | --- | --- | --- | --- | --- | --- | --- | --- | --- | --- | --- | --- | --- | --- | --- |
| ROI | Model | | | | Pain intensity (Scan pain score) | | | | | | Stress (PCL-C total score) | | | | | | Group x Stress | | | | | |  |
|  | *Adj R^2^* | *F* | *df* | *p*-value | *b* | *se* | LLCI | ULCI | *t*-value | *p*-value | *b* | *se* | LLCI | ULCI | *t*-value | *p*-value | *b* | *se* | LLCI | ULCI | *t*-value | *p*-value | |
| **A. All chronic pain conditions** | | |  |  |  |  |  |  |  |  |  |  |  |  |  |  |  |  |  |  |  |  | |
| LNAcc | **0.465** | **7.270** | 2,30 | **<1.92x10-3** | 0.0002 | 0.0042 | -0.0083 | 0.0088 | 0.0562 | 0.9556 | -0.0007 | 0.0008 | -0.0023 | 0.0009 | -0.8755 | 0.3882 | 0.0001 | 0.0004 | -0.0007 | 0.0008 | 0.1454 | 0.8853 | |
| RNAcc | 0.558 | 8.773 | 8,30 | **<1.92x10-3** | 0.0022 | 0.0036 | -0.0052 | 0.0095 | 0.6033 | 0.5508 | **-0.0013** | **0.0006** | **-0.0025** | **-0.0001** | **-2.2219** | **0.0340** | 0.0001 | 0.0002 | -0.0004 | 0.0006 | 0.3171 | 0.7534 | |
| LCaud | 0.385 | 3.137 | 8,30 | **<1.92x10-3** | -0.0058 | 0.0417 | -0.0910 | 0.0794 | -0.1394 | 0.8901 | 0.0012 | 0.0076 | -0.0143 | 0.0167 | 0.1612 | 0.8730 | -0.0023 | 0.0023 | -0.0071 | 0.0024 | -1.0091 | 0.3210 | |
| RCaud | 0.406 | 3.392 | 8,30 | **<1.92x10-3** | -0.0155 | 0.0430 | -0.1034 | 0.0723 | -0.3611 | 0.7206 | -0.0006 | 0.0075 | -0.0159 | 0.0148 | -0.0762 | 0.9398 | -0.0018 | 0.0023 | -0.0064 | 0.0029 | -0.7769 | 0.4433 | |
| LPut | 0.434 | 3.156 | 8,30 | 0.0103 | 0.0056 | 0.0360 | -0.0679 | 0.0791 | 0.1559 | 0.8771 | -0.0009 | 0.0058 | -0.0128 | 0.0111 | -0.1461 | 0.8848 | -0.0012 | 0.0034 | -0.0081 | 0.0057 | -0.3596 | 0.7217 | |
| RPut | 0.494 | 6.114 | 8,30 | **<1.92x10-3** | 0.0213 | 0.0259 | -0.0316 | 0.0743 | 0.8218 | 0.4177 | -0.0037 | 0.0054 | -0.0147 | 0.0072 | -0.6972 | 0.4911 | -0.0027 | 0.0029 | -0.0087 | 0.0033 | -0.9058 | 0.3723 | |
| LPallid | 0.259 | 2.646 | 8,30 | 0.0252 | 0.0090 | 0.0096 | -0.0106 | 0.0286 | 0.9406 | 0.3544 | 0.0002 | 0.0019 | -0.0037 | 0.0041 | 0.1021 | 0.9193 | -0.0012 | 0.0006 | -0.0025 | 0.0001 | -1.9270 | 0.0635 | |
| RPallid | 0.251 | 2.742 | 8,30 | 0.0213 | 0.0049 | 0.0099 | -0.0154 | 0.0252 | 0.4960 | 0.6235 | 0.0000 | 0.0022 | -0.0045 | 0.0044 | -0.0172 | 0.9864 | -0.0011 | 0.0007 | -0.0024 | 0.0003 | -1.5977 | 0.1206 | |
| LAmyg | 0.692 | 13.754 | 8,30 | **<1.92x10-3** | -0.0034 | 0.0053 | -0.0143 | 0.0075 | -0.6410 | 0.5264 | -0.0024 | 0.0014 | -0.0052 | 0.0004 | -1.7820 | 0.0849 | -0.0003 | 0.0004 | -0.0012 | 0.0006 | -0.6718 | 0.5068 | |
| RAmyg | 0.639 | 14.811 | 8,30 | **<1.92x10-3** | -0.0026 | 0.0052 | -0.0133 | 0.0080 | -0.5035 | 0.6183 | -0.0023 | 0.0012 | -0.0048 | 0.0002 | -1.8459 | 0.0748 | -0.0005 | 0.0005 | -0.0015 | 0.0005 | -1.0073 | 0.3219 | |
| LHippo | 0.372 | 4.173 | 8,30 | **<1.92x10-3** | -0.0106 | 0.0251 | -0.0618 | 0.0405 | -0.4249 | 0.6739 | 0.0034 | 0.0072 | -0.0114 | 0.0182 | 0.4733 | 0.6395 | -0.0005 | 0.0023 | -0.0051 | 0.0041 | -0.2206 | 0.8269 | |
| RHippo | 0.448 | 9.892 | 8,30 | **<1.92x10-3** | -0.0151 | 0.0265 | -0.0691 | 0.0390 | -0.5700 | 0.5729 | 0.0042 | 0.0083 | -0.0127 | 0.0211 | 0.5061 | 0.6165 | -0.0009 | 0.0025 | -0.0059 | 0.0041 | -0.3539 | 0.7259 | |
| LThal | 0.222 | 2.793 | 8,30 | 0.0194 | -0.0537 | 0.0543 | -0.1646 | 0.0572 | -0.9890 | 0.3306 | -0.0031 | 0.0132 | -0.0301 | 0.0238 | -0.2367 | 0.8145 | -0.0026 | 0.0043 | -0.0113 | 0.0061 | -0.6140 | 0.5439 | |
| RThal | 0.131 | 1.551 | 8,30 | 0.1818 | -0.0299 | 0.0533 | -0.1387 | 0.0790 | -0.5603 | 0.5794 | -0.0008 | 0.0131 | -0.0275 | 0.0260 | -0.0584 | 0.9538 | 0.0018 | 0.0038 | -0.0060 | 0.0097 | 0.4827 | 0.6328 | |
| LMFC | 0.573 | 12.868 | 8,30 | **<1.92x10-3** | -0.0109 | 0.0155 | -0.0425 | 0.0208 | -0.7006 | 0.4890 | 0.0030 | 0.0039 | -0.0049 | 0.0109 | 0.7796 | 0.4417 | -0.0001 | 0.0018 | -0.0038 | 0.0037 | -0.0439 | 0.9652 | |
| RMFC | 0.569 | 8.247 | 8,30 | **<1.92x10-3** | -0.0028 | 0.0141 | -0.0315 | 0.0260 | -0.1977 | 0.8446 | 0.0039 | 0.0038 | -0.0039 | 0.0118 | 1.0236 | 0.3142 | 0.0003 | 0.0016 | -0.0030 | 0.0037 | 0.2035 | 0.8401 | |
| LAntCgG | 0.521 | 16.437 | 8,30 | **<1.92x10-3** | -0.0203 | 0.0355 | -0.0929 | 0.0522 | -0.5723 | 0.5714 | -0.0070 | 0.0085 | -0.0243 | 0.0103 | -0.8250 | 0.4159 | 0.0011 | 0.0046 | -0.0083 | 0.0104 | 0.2342 | 0.8164 | |
| RAntCgG | 0.472 | 10.908 | 8,30 | **<1.92x10-3** | -0.0138 | 0.0369 | -0.0892 | 0.0615 | -0.3752 | 0.7102 | -0.0066 | 0.0068 | -0.0205 | 0.0074 | -0.9628 | 0.3433 | 0.0033 | 0.0059 | -0.0087 | 0.0152 | 0.5604 | 0.5793 | |
| LMCgG | 0.640 | 10.361 | 8,30 | **<1.92x10-3** | -0.0290 | 0.0290 | -0.0882 | 0.0302 | -1.0009 | 0.3249 | -0.0119 | 0.0063 | -0.0248 | 0.0009 | -1.8930 | 0.0680 | 0.0012 | 0.0050 | -0.0090 | 0.0115 | 0.2417 | 0.8107 | |
| RMCgG | 0.598 | 12.219 | 8,30 | **<1.92x10-3** | -0.0187 | 0.0329 | -0.0859 | 0.0486 | -0.5669 | 0.5750 | -0.0105 | 0.0063 | -0.0234 | 0.0024 | -1.6645 | 0.1064 | 0.0010 | 0.0041 | -0.0073 | 0.0093 | 0.2414 | 0.8109 | |
| LMFG | 0.688 | 10.292 | 8,30 | **<1.92x10-3** | -0.0983 | 0.1059 | -0.3146 | 0.1179 | -0.9285 | 0.3605 | -0.0126 | 0.0226 | -0.0588 | 0.0336 | -0.5588 | 0.5805 | 0.0012 | 0.0102 | -0.0197 | 0.0220 | 0.1126 | 0.9111 | |
| RMFG | 0.714 | 14.455 | 8,30 | **<1.92x10-3** | -0.0140 | 0.1026 | -0.2235 | 0.1954 | -0.1367 | 0.8921 | -0.0213 | 0.0171 | -0.0563 | 0.0137 | -1.2437 | 0.2232 | -0.0025 | 0.0097 | -0.0223 | 0.0172 | -0.2600 | 0.7966 | |
| LAIns | 0.495 | 3.939 | 8,30 | 0.0028 | 0.0122 | 0.0396 | -0.0688 | 0.0931 | 0.3069 | 0.7610 | -0.0001 | 0.0067 | -0.0138 | 0.0136 | -0.0095 | 0.9925 | -0.0012 | 0.0038 | -0.0088 | 0.0065 | -0.3152 | 0.7548 | |
| RAIns | 0.487 | 3.088 | 8,30 | 0.0116 | 0.0105 | 0.0342 | -0.0593 | 0.0804 | 0.3074 | 0.7607 | -0.0015 | 0.0069 | -0.0156 | 0.0126 | -0.2192 | 0.8280 | -0.0011 | 0.0031 | -0.0074 | 0.0052 | -0.3654 | 0.7174 | |
| LPIns | 0.656 | 16.110 | 8,30 | **<1.92x10-3** | -0.0145 | 0.0133 | -0.0417 | 0.0127 | -1.0883 | 0.2851 | -0.0036 | 0.0033 | -0.0103 | 0.0030 | -1.1109 | 0.2755 | -0.0006 | 0.0011 | -0.0029 | 0.0017 | -0.5441 | 0.5904 | |
| RPIns | 0.720 | 25.272 | 8,30 | **<1.92x10-3** | -0.0112 | 0.0138 | -0.0394 | 0.0169 | -0.8133 | 0.4225 | -0.0048 | 0.0028 | -0.0105 | 0.0009 | -1.7132 | 0.0970 | -0.0006 | 0.0010 | -0.0026 | 0.0015 | -0.5619 | 0.5784 | |
|  |  |  |  |  |  |  |  |  |  |  |  |  |  |  |  |  |  |  |  |  |  |  | |
|  | | |  |  |  |  |  |  |  |  |  |  |  |  |  |  |  |  |  |  |  |  | |
| LNAcc | 0.476 | 3.095 | 8,17 | 0.0239 | -0.0001 | 0.0058 | -0.0123 | 0.0120 | -0.0229 | 0.9820 | -0.0011 | 0.0014 | -0.0041 | 0.0019 | -0.7710 | 0.4513 | 0.0004 | 0.0008 | -0.0014 | 0.0021 | 0.4178 | 0.6813 | |
| RNAcc | 0.480 | 1.528 | 8,17 | 0.2198 | 0.0024 | 0.0042 | -0.0064 | 0.0111 | 0.5696 | 0.5764 | -0.0015 | 0.0013 | -0.0042 | 0.0012 | -1.1889 | 0.2508 | 0.0002 | 0.0005 | -0.0009 | 0.0012 | 0.3158 | 0.7560 | |
| LCaud | 0.392 | 3.009 | 8,17 | 0.0268 | -0.0137 | 0.0429 | -0.1042 | 0.0768 | -0.3195 | 0.7532 | 0.0057 | 0.0137 | -0.0232 | 0.0346 | 0.4166 | 0.6822 | -0.0021 | 0.0053 | -0.0133 | 0.0092 | -0.3858 | 0.7045 | |
| RCaud | 0.373 | 1.523 | 8,17 | 0.2213 | -0.0212 | 0.0453 | -0.1167 | 0.0743 | -0.4681 | 0.6457 | 0.0036 | 0.0151 | -0.0282 | 0.0354 | 0.2401 | 0.8131 | -0.0018 | 0.0049 | -0.0121 | 0.0085 | -0.3635 | 0.7207 | |
| LPut | 0.250 | 1.178 | 8,17 | 0.3667 | 0.0103 | 0.0409 | -0.0760 | 0.0965 | 0.2511 | 0.8048 | 0.0041 | 0.0111 | -0.0194 | 0.0275 | 0.3653 | 0.7194 | 0.0023 | 0.0061 | -0.0105 | 0.0151 | 0.3804 | 0.7083 | |
| RPut | 0.339 | 5.444 | 8,17 | 0.0017 | 0.0160 | 0.0297 | -0.0467 | 0.0786 | 0.5382 | 0.5974 | -0.0033 | 0.0109 | -0.0264 | 0.0198 | -0.3042 | 0.7647 | -0.0014 | 0.0049 | -0.0118 | 0.0091 | -0.2752 | 0.7865 | |
| LPallid | 0.210 | 1. 748 | 8,17 | 0.1585 | 0.0078 | 0.0127 | -0.0190 | 0.0346 | 0.6126 | 0.5483 | 0.0006 | 0.0034 | -0.0067 | 0.0078 | 0.1617 | 0.8735 | -0.0014 | 0.0011 | -0.0037 | 0.0008 | -1.3524 | 0.1940 | |
| RPallid | 0.394 | 1.637 | 8,17 | 0.1867 | 0.0038 | 0.0124 | -0.0223 | 0.0299 | 0.3072 | 0.7624 | 0.0008 | 0.0041 | -0.0078 | 0.0094 | 0.1941 | 0.8484 | -0.0019 | 0.0012 | -0.0044 | 0.0006 | -1.6179 | 0.1241 | |
| LAmyg | 0.616 | 3.030 | 8,17 | 0.0261 | 0.0020 | 0.0081 | -0.0152 | 0.0191 | 0.2409 | 0.8125 | -0.0031 | 0.0018 | -0.0068 | 0.0007 | -1.7402 | 0.0999 | -0.0008 | 0.0011 | -0.0031 | 0.0016 | -0.6875 | 0.5010 | |
| RAmyg | 0.556 | 2.233 | 8,17 | 0.0781 | 0.0780 | 0.0780 | 0.0780 | 0.0780 | 0.0780 | 0.0780 | -0.0035 | 0.0021 | -0.0079 | 0.0008 | -1.7184 | 0.1039 | -0.0007 | 0.0014 | -0.0037 | 0.0022 | -0.5297 | 0.6032 | |
| LHippo | 0.428 | 2.570 | 8,17 | 0.0485 | 0.0028 | 0.0357 | -0.0725 | 0.0782 | 0.0792 | 0.9378 | -0.0025 | 0.0105 | -0.0247 | 0.0196 | -0.2403 | 0.8129 | -0.0005 | 0.0050 | -0.0110 | 0.0099 | -0.1064 | 0.9165 | |
| RHippo | 0.468 | 2.139 | 8,17 | 0.0894 | -0.0007 | 0.0487 | -0.1034 | 0.1021 | -0.0134 | 0.9895 | -0.0031 | 0.0114 | -0.0271 | 0.0209 | -0.2745 | 0.7870 | -0.0012 | 0.0070 | -0.0160 | 0.0136 | -0.1710 | 0.8662 | |
| LThal | 0.144 | 0.961 | 8,17 | 0.4960 | 0.0188 | 0.0724 | -0.1339 | 0.1714 | 0.2592 | 0.7986 | -0.0113 | 0.0197 | -0.0529 | 0.0303 | -0.5725 | 0.5745 | -0.0060 | 0.0090 | -0.0249 | 0.0129 | -0.6714 | 0.5110 | |
| RThal | -0.188 | 0.359 | 8,17 | 0.9280 | 0.0291 | 0.0683 | -0.1149 | 0.1732 | 0.4265 | 0.6751 | -0.0099 | 0.0218 | -0.0558 | 0.0360 | -0.4535 | 0.6559 | 0.0004 | 0.0063 | -0.0129 | 0.0136 | 0.0569 | 0.9553 | |
| LMFC | 0.537 | 4.936 | 8,17 | 0.0028 | -0.0142 | 0.0236 | -0.0639 | 0.0355 | -0.6042 | 0.5537 | 0.0016 | 0.0076 | -0.0145 | 0.0177 | 0.2079 | 0.8378 | -0.0011 | 0.0037 | -0.0089 | 0.0066 | -0.3085 | 0.7615 | |
| RMFC | 0.541 | 4.452 | 8,17 | 0.0047 | -0.0019 | 0.0263 | -0.0573 | 0.0535 | -0.0723 | 0.9432 | 0.0047 | 0.0080 | -0.0122 | 0.0215 | 0.5853 | 0.5660 | 0.0000 | 0.0047 | -0.0100 | 0.0100 | 0.0087 | 0.9931 | |
| LAntCgG | 0.357 | 12.174 | 8,17 | **<1.92x10-3** | -0.0222 | 0.0629 | -0.1549 | 0.1106 | -0.3525 | 0.7288 | -0.0100 | 0.0211 | -0.0546 | 0.0345 | -0.4744 | 0.6413 | 0.0008 | 0.0126 | -0.0257 | 0.0273 | 0.0614 | 0.9517 | |
| RAntCgG | 0.538 | 4.393 | 8,17 | 0.0050 | -0.0146 | 0.0485 | -0.1170 | 0.0878 | -0.3012 | 0.7669 | -0.0015 | 0.0171 | -0.0377 | 0.0346 | -0.0899 | 0.9294 | 0.0102 | 0.0102 | -0.0113 | 0.0317 | 1.0016 | 0.3306 | |
| LMCgG | 0.545 | 12.175 | 8,17 | **<1.92x10-3** | -0.0164 | 0.0457 | -0.1130 | 0.0801 | -0.3593 | 0.7238 | -0.0129 | 0.0151 | -0.0446 | 0.0189 | -0.8554 | 0.4042 | 0.0045 | 0.0098 | -0.0162 | 0.0252 | 0.4604 | 0.6511 | |
| RMCgG | 0.472 | 3.279 | 8,17 | 0.0189 | -0.0232 | 0.0494 | -0.1275 | 0.0811 | -0.4687 | 0.6452 | -0.0059 | 0.0163 | -0.0404 | 0.0285 | -0.3641 | 0.7203 | 0.0018 | 0.0090 | -0.0171 | 0.0207 | 0.1994 | 0.8443 | |
| LMFG | 0.786 | 19.297 | 8,17 | **<1.92x10-3** | -0.0453 | 0.1105 | -0.2786 | 0.1879 | -0.4101 | 0.6868 | -0.0396 | 0.0331 | -0.1094 | 0.0302 | -1.1964 | 0.2480 | 0.0128 | 0.0146 | -0.0180 | 0.0436 | 0.8768 | 0.3928 | |
| RMFG | 0.641 | 33.446 | 8,17 | **<1.92x10-3** | 0.0189 | 0.1307 | -0.2568 | 0.2947 | 0.1449 | 0.8865 | -0.0415 | 0.0404 | -0.1268 | 0.0438 | -1.0269 | 0.3189 | 0.0069 | 0.0165 | -0.0279 | 0.0418 | 0.4209 | 0.6791 | |
| LAIns | 0.310 | 1.931 | 8,17 | 0.1211 | 0.0385 | 0.0645 | -0.0975 | 0.1746 | 0.5973 | 0.5582 | -0.0037 | 0.0111 | -0.0271 | 0.0198 | -0.3286 | 0.7465 | 0.0025 | 0.0090 | -0.0166 | 0.0215 | 0.2729 | 0.7882 | |
| RAIns | 0.237 | 1.652 | 8,17 | 0.1827 | 0.0307 | 0.0333 | -0.0396 | 0.1010 | 0.9216 | 0.3696 | -0.0081 | 0.0099 | -0.0290 | 0.0127 | -0.8225 | 0.4222 | 0.0012 | 0.0042 | -0.0076 | 0.0100 | 0.2893 | 0.7758 | |
| LPIns | 0.539 | 3.117 | 8,17 | 0.0232 | -0.0117 | 0.0204 | -0.0547 | 0.0314 | -0.5712 | 0.5753 | -0.0052 | 0.0068 | -0.0195 | 0.0091 | -0.7698 | 0.4520 | -0.0008 | 0.0023 | -0.0056 | 0.0039 | -0.3684 | 0.7171 | |
| RPIns | 0.673 | 4.112 | 8,17 | 0.0069 | -0.0041 | 0.0191 | -0.0444 | 0.0361 | -0.2171 | 0.8307 | -0.0031 | 0.0059 | -0.0156 | 0.0093 | -0.5325 | 0.6013 | -0.0009 | 0.0026 | -0.0063 | 0.0045 | -0.3589 | 0.7241 | |
| ROI: region of interest; PCLC: posttraumatic stress disorder checklist - civilian; L/R: left/right; NAcc: nucleus accumbens; Caud: caudate nucleus; Put: putamen; Pallid: pallidum; Amyg: amygdala; Hippo: hippocampus; Thal: thalamus proper; MFC: medial frontal cortex; AntCgG: anterior cingulate gyrus; MCgG: middle cingulate gyrus; MFG: middle frontal gyrus; AIns: anterior insula; Pins: posterior insula; Adj R^2^: adjusted coefficient of determination; se: standard error; LLCI: bootstrapped 95% lower levels confidence interval; ULCI: bootstrapped 95% upper levels confidence interval  Statistically significant associations (*p*<0.05 within each model) are in bold and highlighted in grey | | | | | | | | | | | | | | | | | | | | | | | |

| **Table S7.** Results of the analyses comparing people with chronic pain using pain-related medication to people with chronic pain not using pain-related medication | | | | | | |
| --- | --- | --- | --- | --- | --- | --- |
| **ROI** | *b* | *se* | LLCI | ULCI | *t*-value | *p*-value |
| LNAcc | -0.0044 | 0.0094 | -0.0234 | 0.0147 | -0.4611 | 0.6469 |
| RNAcc | -0.0031 | 0.0096 | -0.0224 | 0.0163 | -0.3195 | 0.7508 |
| LCaud | -0.0315 | 0.0864 | -0.2053 | 0.1424 | -0.3642 | 0.7174 |
| RCaud | -0.0816 | 0.0921 | -0.2669 | 0.1037 | -0.8860 | 0.3802 |
| LPut | -0.0712 | 0.1034 | -0.2794 | 0.1370 | -0.6881 | 0.4948 |
| RPut | -0.0684 | 0.1041 | -0.2779 | 0.1410 | -0.6575 | 0.5141 |
| LPallid | 0.0210 | 0.0271 | -0.0336 | 0.0755 | 0.7734 | 0.4432 |
| RPallid | 0.0221 | 0.0288 | -0.0358 | 0.0800 | 0.7676 | 0.4466 |
| LAmyg | 0.0025 | 0.0185 | -0.0347 | 0.0397 | 0.1347 | 0.8934 |
| RAmyg | -0.0019 | 0.0172 | -0.0366 | 0.0328 | -0.1108 | 0.9122 |
| LHippo | -0.0214 | 0.0773 | -0.1770 | 0.1342 | -0.2767 | 0.7833 |
| RHippo | -0.0338 | 0.0799 | -0.1947 | 0.1271 | -0.4228 | 0.6744 |
| LThal | -0.1391 | 0.1536 | -0.4483 | 0.1701 | -0.9057 | 0.3698 |
| RThal | -0.2072 | 0.1558 | -0.5208 | 0.1064 | -1.3301 | 0.1901 |
| LMFC | -0.0076 | 0.0567 | -0.1217 | 0.1065 | -0.1334 | 0.8945 |
| RMFC | 0.0036 | 0.0496 | -0.0962 | 0.1033 | 0.0719 | 0.9430 |
| LAntCgG | -0.0506 | 0.1153 | -0.2826 | 0.1814 | -0.4389 | 0.6628 |
| RAntCgG | -0.0544 | 0.1011 | -0.2580 | 0.1492 | -0.5379 | 0.5932 |
| LMCgG | -0.0762 | 0.0998 | -0.2771 | 0.1247 | -0.7637 | 0.4490 |
| RMCgG | -0.0876 | 0.1056 | -0.3003 | 0.1250 | -0.8295 | 0.4111 |
| LMFG | 0.1800 | 0.3361 | -0.4965 | 0.8565 | 0.5356 | 0.5948 |
| RMFG | 0.3914 | 0.2863 | -0.1849 | 0.9677 | 1.3672 | 0.1782 |
| LAIns | -0.0105 | 0.0788 | -0.1691 | 0.1481 | -0.1327 | 0.8950 |
| RAIns | 0.0788 | 0.0896 | -0.1015 | 0.2592 | 0.8797 | 0.3836 |
| LPIns | 0.0606 | 0.0399 | -0.0198 | 0.1410 | 1.5180 | 0.1359 |
| RPIns | 0.0642 | 0.0394 | -0.0150 | 0.1435 | 1.6309 | 0.1097 |
| ROI: region of interest; PCLC: posttraumatic stress disorder checklist - civilian; L/R: left/right; NAcc: nucleus accumbens; Caud: caudate nucleus; Put: putamen; Pallid: pallidum; Amyg: amygdala; Hippo: hippocampus; Thal: thalamus proper; MFC: medial frontal cortex; AntCgG: anterior cingulate gyrus; MCgG: middle cingulate gyrus; MFG: middle frontal gyrus; AIns: anterior insula; Pins: posterior insula; Adj R^2^: adjusted coefficient of determination; se: standard error; LLCI: bootstrapped 95% lower levels confidence interval; ULCI: bootstrapped 95% upper levels confidence interval  Statistically significant associations (*p*<0.05 within each model) are in bold and highlighted in grey | | | | | | |
